# Dietary Methanol Regulates Human Gene Activity

**DOI:** 10.1371/journal.pone.0102837

**Published:** 2014-07-17

**Authors:** Anastasia V. Shindyapina, Igor V. Petrunia, Tatiana V. Komarova, Ekaterina V. Sheshukova, Vyacheslav S. Kosorukov, Gleb I. Kiryanov, Yuri L. Dorokhov

**Affiliations:** 1 A. N. Belozersky Institute of Physico-Chemical Biology, Moscow State University, Moscow, Russia; 2 N. I. Vavilov Institute of General Genetics, Russian Academy of Science, Moscow, Russia; 3 Blokhin Cancer State Research Center, Russian Academy of Medical Sciences, Moscow, Russia; University of Saarland Medical School, Germany

## Abstract

Methanol (MeOH) is considered to be a poison in humans because of the alcohol dehydrogenase (ADH)-mediated conversion of MeOH to formaldehyde (FA), which is toxic. Our recent genome-wide analysis of the mouse brain demonstrated that an increase in endogenous MeOH after ADH inhibition led to a significant increase in the plasma MeOH concentration and a modification of mRNA synthesis. These findings suggest endogenous MeOH involvement in homeostasis regulation by controlling mRNA levels. Here, we demonstrate directly that study volunteers displayed increasing concentrations of MeOH and FA in their blood plasma when consuming citrus pectin, ethanol and red wine. A microarray analysis of white blood cells (WBC) from volunteers after pectin intake showed various responses for 30 significantly differentially regulated mRNAs, most of which were somehow involved in the pathogenesis of Alzheimer's disease (AD). There was also a decreased synthesis of hemoglobin mRNA, *HBA* and *HBB*, the presence of which in WBC RNA was not a result of red blood cells contamination because erythrocyte-specific marker genes were not significantly expressed. A qRT-PCR analysis of volunteer WBCs after pectin and red wine intake confirmed the complicated relationship between the plasma MeOH content and the mRNA accumulation of both genes that were previously identified, namely, *GAPDH* and *SNX27*, and genes revealed in this study, including *MME*, *SORL1*, *DDIT4*, *HBA* and *HBB*. We hypothesized that human plasma MeOH has an impact on the WBC mRNA levels of genes involved in cell signaling.

## Introduction

Robert Boyle first described wood spirits, or methanol (MeOH), as the “sowrish spirit” of boxwood pyrolysis in 1661 [1], and its function in plant and animal life has since been unclear. In higher plants, cell wall pectin methylesterase (PME) produces MeOH by pectin demethylation [2]–[9]. Terrestrial atmospheric MeOH emissions come from volcanoes, H_2_ and CO_2_ generation within seafloor hydrothermal systems [10]–[12] and biomass combustion. However PME-mediated emissions from plants are most likely the largest source of MeOH in the atmosphere [13]–[15]. MeOH accumulates in a plant's intercellular air space or liquid pool at night when the stomata close, and a large MeOH release can be observed in the morning when the stomata open [8], [16], [17]. Gaseous MeOH was traditionally considered to be a biochemical “waste product” [8], [9]. However, the effects of PME-generated MeOH from plants-emitters on plants-receivers’ were recently studied [2]. These investigations demonstrated that increased MeOH emissions from PME-transgenic or mechanically wounded non-transgenic plants retard the growth of bacterial pathogen *Ralstonia solanacearum* in neighboring “receiver” plants. This antibacterial resistance is accompanied by the up-regulation of genes that control stress and cell-to-cell communication in the “receiver”. These results suggest that MeOH is a signaling molecule for within-plant and plant-to-plant communications [2].

In humans, MeOH is considered to be a poison because alcohol dehydrogenases (ADHs) can metabolize MeOH into toxic formaldehyde (FA) [18]–[27]. Then FA is oxidized to *S*-formylglutathione in a reaction that requires reduced glutathione and is mediated by a NAD-dependent FA dehydrogenase or alcohol dehydrogenase 3 (ADH3) [24], [28]. In the next step, thiolase catalyzes the conversion of *S*-formylglutathione to formic acid, which dissociates to produce formate and a hydrogen ion. The third reaction (formate to CO_2_ and water) is catalyzed by catalase through a combination with tetrahydrofolate to produce 10-formyl tetrahydrofolate [21], [25], [29]. FA did not accumulate substantially in MeOH-intoxicated humans [29], [30]. Moreover, FA was not detected in blood, urine, or tissues obtained from MeOH-treated animals, and humans poisoned with the alcohol did not have increased FA [22], [24], [30]–[34]. The time needed to semi-transform FA into formic acid was 1–2 minutes in many species, including humans [35], [36]. Recent data have indicated that MeOH and short-lived FA are natural compounds in normal, healthy human individuals [3], [35], [37]–[43]. The endogenous MeOH content was more than 400 times lower than harmful concentrations [44]. Over 50 mg of FA is produced and metabolized in an adult human body every day according to calculations, and an adult human liver metabolize 22 mg of FA per minute [45]. Its content (0.083±0.001 mM) may be detected in the urine of healthy people [46]. The increased production of endogenous FA was recently shown to be a possible marker for progressive senile dementia [46]. The origin of endogenous MeOH in humans has not yet been elucidated, but two sources of this MeOH have been suggested [37], namely, anaerobic fermentation by gut bacteria [47] and certain metabolic processes in which *S*-adenosyl methionine (SAM) may be transformed into MeOH [48], [49]. SAM is a universal endogenous methyl donor for various methylation reactions, including the methylation of proteins, phospholipids, DNA, RNA and other molecules that take part in the basic mechanisms of epigenetic phenomena. Genome-wide methylation analysis has indicated DNA methylation profiles that are specific for aging and longevity [50]. Moreover, the unmasked DNA methylation landscape shows that DNA obtained from a 103-y-old donor was more unmethylated overall than DNA from the same cell type in a neonate [51]. Differentially methylated genes are strikingly enriched with loci associated with neurological disorders, psychological disorders, and cancers [52]. Protein carboxylmethylation involves the methylation of the COOH group in amino acids, and the reaction is catalyzed by methyltransferases [53]. The carboxyl methyl ester products readily hydrolyze and produce MeOH under neutral and basic pH conditions or by methylesterase [54]–[58]. Protein carboxymethylase is highly localized in the brain and pituitary gland of several mammalian species [54]–[56]. Interestingly, aspartame, which is a widely used synthetic non-nutritive sweetener, is the methyl ester of a dipeptide (*N*-*L*-*α*-aspartyl-*L*-phenylalanine) that is likely to convert to MeOH with the participation of protein methylesterases [35], [59], [60].

In considering the function of MeOH, it is important to estimate the toxic consequences of exogenous MeOH intake and the production of endogenous MeOH in humans. Because ADHs evolve utilizing MeOH and ethanol (EtOH), EtOH functions as a powerful competitive inhibitor at low concentrations [21]. The enzyme has a strong preference for converting EtOH to acetaldehyde over the conversion of MeOH to FA [61]. A detection of EtOH and MeOH in breaths from the same volunteer cohort suggested that MeOH and EtOH are formed in the body from different substances and*/*or processes [41]. EtOH protection from FA production may explain the U-shaped curve that describes dependence between alcohol consumption and cardiovascular diseases [62]–[64]. Very low levels of EtOH in the bloodstream would prevent FA production from endogenous and dietary MeOH in humans in accordance with the clinical practice of when to inhibit by EtOH metabolite production after MeOH poisoning [65], [66]. EtOH can be replaced with 4-methylpyrazole (4-MP) as a potent inhibitor of ADH activity because 4-MP has a longer duration of action and apparently fewer adverse effects [35], [67], [68]. To test the role of ADH in maintaining a low MeOH concentration, we recently showed that the intraperitoneal administration of 4-MP resulted in a significant increase MeOH, EtOH and FA concentrations in mouse plasma [47]. Removing the intestine significantly decreased the addition of MeOH to the plasma suggested the gut flora may be involved in endogenous MeOH production. Increased MeOH and EtOH contents in the liver homogenate were observed after 4-MP administration into the portal vein. Thus the ADH in the liver was confirmed as the primary enzyme for metabolizing MeOH. Liver mRNA quantification showed changes in the accumulation of mRNA from genes involved in cell signaling and detoxification processes. Endogenous MeOH has been hypothesized to act as a homeostatic regulator by controlling mRNA synthesis [47].

4-MP intake by healthy women and men also resulted in the significant elevation of endogenous EtOH and MeOH in plasma [69], indicating a high level of MeOH generated by endogenous human sources. These data raise a question whether MeOH is a metabolic waste product or a chemical with specific functions in humans.

Human MeOH-responsive genes (MRGs) were recently identified [3]. The MRGs were discovered in exposed to MeOH HeLa cells lacking ADH [70]–[72], thereby eliminating from the analysis any confounding effects from genes involved in FA and formic acid detoxification. MeOH that is generated by the pectin/PME complex in the gastrointestinal tract of mice induces MRG mRNA regulated accumulation in brain. Mice prefer the odor of MeOH to the odors of other plant volatiles, and MeOH exposure alters MRG mRNA accumulation in the mouse brain. This finding led to the conclusion that the MeOH emitted by wounded plants may have a role in plant-animal signaling. Among the four identified MRGs, the *glyceraldehyde 3-phosphate dehydrogenase (GAPDH)* and *sorting nexin 27 (SNX27)* of mouse brains exhibited high responsiveness to MeOH intake or pectin/PME complex [pectin(PME+)] ingestion. *GAPDH* has often been referred to as a “housekeeping” gene and is used to standardize northern blots. However, a number of novel functions for GAPDH beyond glycolysis have been described over the last two decades, including nuclear trafficking, Aβ biogenesis and AD-related apoptosis [73]–[76]. A putative signaling function for MeOH was supported by the simultaneous increase in *mSNX27* mRNA synthesis. Recent data showed the involvement of SNX27 in signal transmission [77], [78] and AD pathogenesis [79], [80]. We suggested that plant pectin and PME-generated MeOH could be one of the plant compounds that reduce progress of AD [3].

However, we found complex and unpredictable behavior testing the transcriptional activity of these genes in the WBCs of volunteers who ate the portion of salad. This finding is apparently explained by the presence in the blood plasma of a mixture of MeOH and its metabolites, which is to say that human blood plasma MeOH is converted to FA and formic acid by the ADH and aldehyde dehydrogenase (AlDH) of the liver. Therefore, human WBCs may not only be exposed to MeOH but also to its metabolic products. Thus, the reaction of the transcriptome can be more complex and not limited to the four identified genes.

In the present study, we estimated the level of plasma MeOH production by endogenous sources. Moreover, we conducted whole genome analysis, which revealed the gene responses of healthy volunteer WBCs at the transcriptome level through human genome microarrays. We investigated the gene expression changes between WBC samples of fasted volunteers before and after citrus pectin intake and showed 32 significantly differentially regulated mRNAs. We concluded that human plasma MeOH, which is replenished from endogenous and exogenous sources, has an impact on the WBC mRNA levels of the genes involved in cell signaling.

## Results

### Pectin and alcohol intake increases MeOH and FA contents in human blood plasma

**Figure 1:**
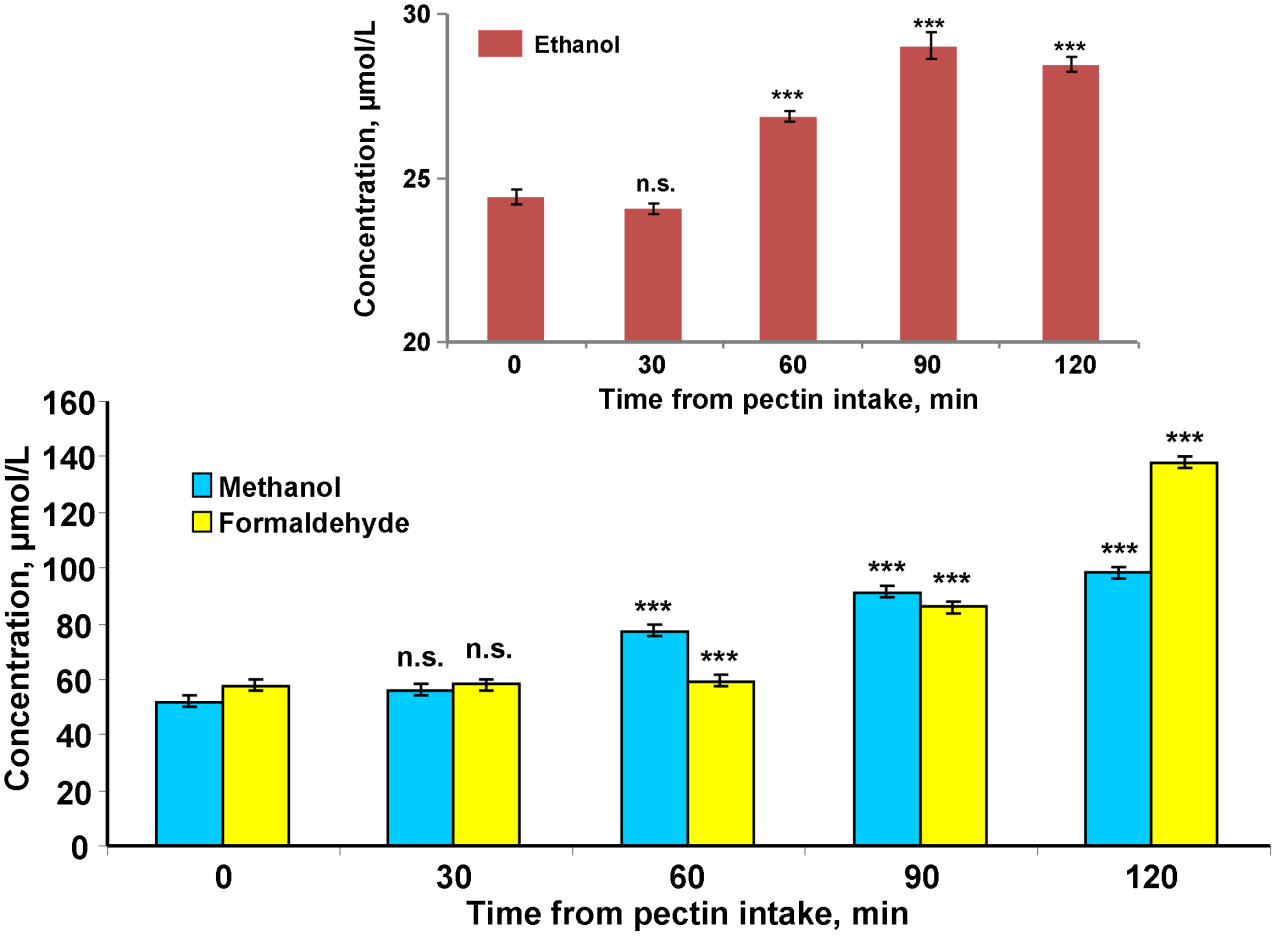
MeOH appearance in human blood is accompanied by the formation of FA and EtOH molecules. The dynamics of MeOH and FA changes in blood plasma after administering pectin are shown. Each volunteer took capsules containing citrus pectin (6 g). After 30, 60, 90 and 120 min, blood samples were obtained and analyzed for MeOH and FA content by GC and HPLC, respectively. The inset shows the ethanol concentrations after pectin administration. The standard error bars are indicated. ***P<0.001 (Student’s *t*-test, n = 8); n.s., not significantly different.

Human endogenic MeOH is thought to be rapidly consumed during renal and pulmonary excretion and the chain of metabolic transformations involving ADH and AlDH [18]–[27]. FA was considered to be the first toxic product of MeOH metabolic clearance. It is a short-lived molecule that vanishes rapidly in normal human blood [25], [35], [36]. We investigated the conversion dynamics of MeOH to FA regarding the increase the MeOH content in the volunteers’ blood after administering a small amount of citrus pectin, which increases the MeOH content in mouse blood [3]. Figure 1 shows that the MeOH content of the blood increases markedly at 1 hour after citrus pectin intake. The initial FA content was comparable to the MeOH and increased over the two hours of observation. Along with blood from the cubital vein, we selected saliva, which is easily obtained through noninvasive means and thus has great potential for screening large cohorts [81]. Figure S1 shows that the MeOH content in saliva was 20% lower on average than it was in the blood, but the concentration also increased with time.

It can be assumed that the ADH-mediated increase in the blood FA content should not affect the endogenous EtOH content because MeOH can displace ADH molecules from the ethanol-to-acetaldehyde conversion. Indeed, volunteers who received citrus pectin exhibited a small but statistically significant increase in EtOH according to the results shown in Figure 1 (inset).

**Figure 2:**
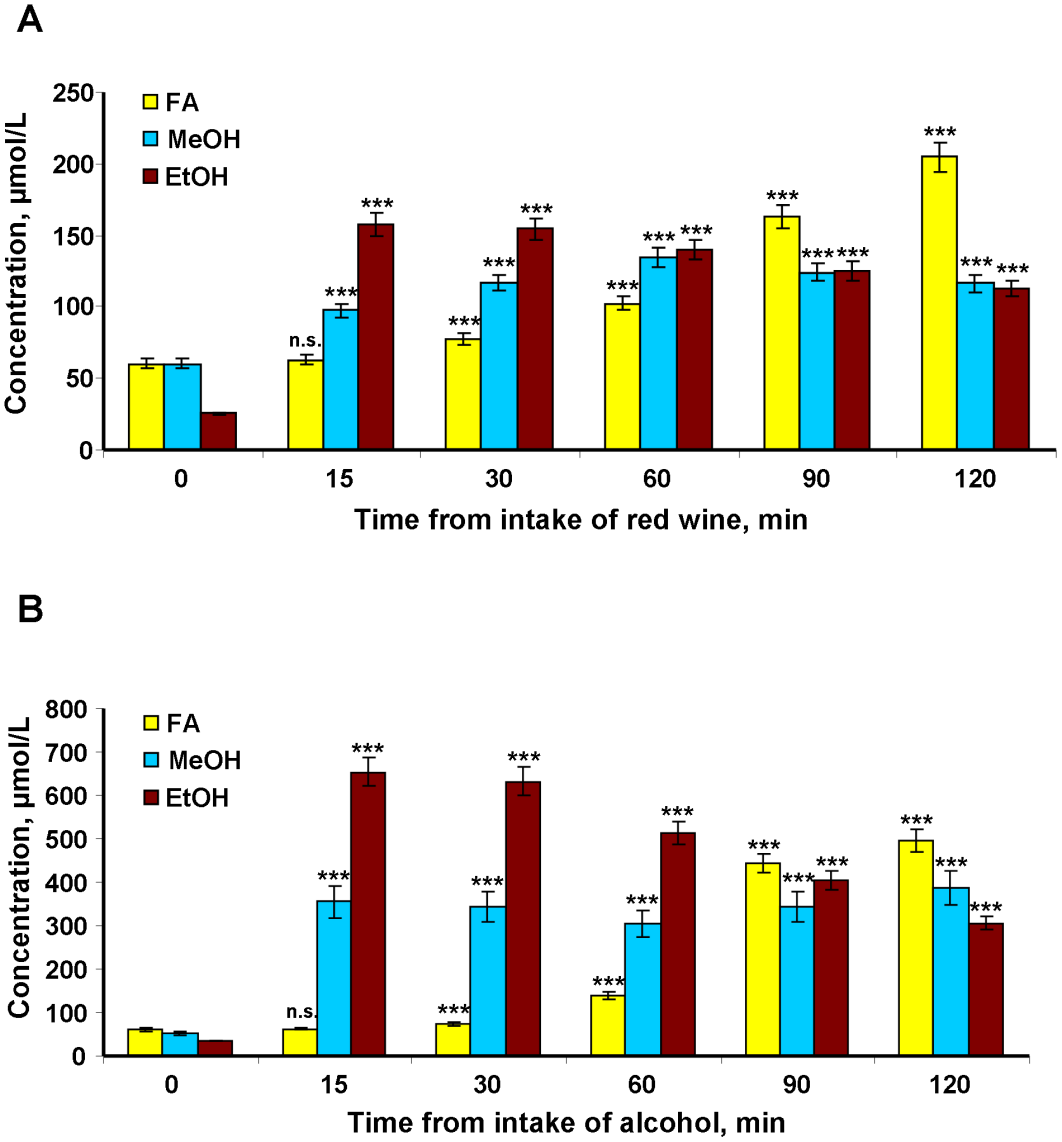
The MeOH, EtOH and FA content of human blood plasma after red wine and alcohol intake. Each of the eight volunteers drank 150(13.731% EtOH and 0.0424% MeOH content, respectively) (A) or 40% v/v ethanol (1 ml per kg body weight) (B). After 15, 30, 60, 90 and 120 min, blood samples were collected and analyzed for MeOH, EtOH and FA contents by GC and HPLC, respectively. The data are presented as the means ± SE. Student’s *t*-test *P*-values were evaluated to determine the statistical significance of the MeOH, EtOH and FA content differences before and after alcohol intake. ***P<0.001 (Student’s *t*-test, n = 8); n.s., not significantly different.

Next, we examined the influence of red wine intake on human plasma MeOH. Red wine is a plant product and source of antioxidants, and it is now regarded as a dietary and preventive means of reducing the risk of cardiovascular and neurodegenerative diseases in humans [82], [83]. However, red wine may influence the MeOH content of the blood. To evaluate the role of red wine as an alcoholic beverage in the human MeOH balance, we evaluated volunteers who drank a glass of red wine (150 ml). A chromatographic analysis of the drink testified that it contained 13.731% EtOH (Fig. S2) and 0.0424% MeOH (Fig. S3). After the volunteers received red wine, blood and saliva were collected and the samples were analyzed by gas chromatography (GC). The MeOH content of the blood (Fig. 2A) and saliva (Fig. S4) increased at 15 minutes after administering the red wine. The increase continued an hour, and then there was a smooth drop in the MeOH content until 120 minutes of observation. Conversely, the amount of FA increased to 90–120 minutes when the MeOH and EtOH level fell (Fig. 2A). The effect of drinking red wine on the MeOH balance is difficult to calculate. On one hand, drinking wine introduces exogenous MeOH (63.6 mg in 150 ml); on the other hand, wine EtOH can increase the pool of endogenous MeOH. Our calculations show that the MeOH contribution from wine was an essential part of its increase in the blood. However, we allowed for the contribution of endogenous MeOH sources because the EtOH in wine can "block" ADH and reduce the "flow" of endogenous MeOH. The accelerated increase in FA levels during the second hour after taking red wine also suggests the involvement of an endogenous pool of MeOH. Endogenous sources of MeOH are not known, but we can assume a high level of production because the MeOH content in the blood is determined primarily by its transformation to FA (metabolic clearance) and also through MeOH removal from the blood by renal and pulmonary excretion. Evaluating the impact of endogenous sources in the MeOH production is possible when the oxidation of MeOH is reduced during ADH competitive inhibition with EtOH. Experiments with volunteers after EtOH intake can assess lower-level MeOH production by endogenous sources because it is difficult to imagine the complete removal of ADH molecules from MeOH metabolism. To conduct this experiment, we selected a group of volunteers (4 males and 4 females) weighing 47 to 90 kg and aged 21 to 67 years. Each volunteer was drinking alcohol on an empty stomach at a volume of 1 ml of 40% EtOH per 1 kg of weight. The alcohol was free of MeOH, as evidenced by GC (Fig. S5). Figure 2B shows that drinking alcohol resulted in increase EtOH and a rapid and almost 6.5-fold increase in plasma MeOH content from 53.3±2.4 to 345.4±49.9 µmol/L in the first 15 min after alcohol administration. The MeOH concentration then remained constant with a slight decrease at the 60-minute point. If we compare these results separately for men and women, a gender difference becomes clear. In women, there was a rise in blood of MeOH during the first 15 minutes, and it increased faster and at a steeper slope than it did in men. Therefore, the MeOH concentration at the 60-minute point was more noticeable but was not statistically significant (Fig. S6). The FA content in the blood of volunteers who drank 40% EtOH increased slowly during the first 60 minutes, rapidly over the next 30 minutes and reached a level of 1.11 mM (33.3 mg/l) at the 120^th^ minute.

We concluded that pectin intake is inevitably accompanied by a marked increase in blood MeOH and toxic FA. Moreover, displacing ADH from MeOH metabolism with alcohol can cause the rapid release of essential amounts of endogenous MeOH and then FA into the blood.

### A genome-wide analysis of human WBCs after the ingestion of citrus pectin

To understand the role of endogenous and dietary MeOH in humans, we conducted a full-genome analysis of WBCs in volunteers after administering pectin as a MeOH-generating substance. The WBC transcripts of 18 volunteers who received citrus pectin were analyzed on the Human HT-12 v.4. Expression BeadChip arrays by targeting more than 25,000 annotated genes with more than 48,000 probes. Significant gene expression differences between the samples after pectin intake and the controls were visualized in a cluster diagram (Fig. S7). We identified transcripts that were expressed with detection *q*-values <0.05 as follows: 106 were up-regulated (Table S1) and 17 were down-regulated (Table S2). Among these results, we selected the genes with the highest gene expression differences (Table 1). Interestingly, (i) there were no detected mRNAs for ADH and NAD-dependent FA dehydrogenase, genes that are involved in the MeOH “detoxifying” metabolism, (ii) most of the identified genes were somehow involved in AD pathogenesis, and (iii) the decreased synthesis of hemoglobin mRNA, *HBA* and *HBB*, was revealed. The presence of hemoglobin mRNAs in the WBCs was not a result of contamination with red blood cells because erythrocyte-specific marker genes [107], [108], *GYPA*, *EBP4*, *SPTA* and *ALAS2*, did not show significant changes. Thus the decreased HBA1 and HBB expression in WBCs did not result from erythropoiesis but from a different mechanism.

**Table 1:** List of significantly down- and up-regulated genes in the WBCs of volunteers after citrus pectin intake.

Gene symbol	Gene description	Genome location	Accession number	*q*-value	Fold change vs. control	Gene function (from KEGG* and NCBI**)
**Down-regulated genes**						
DDIT4&	DNA-damage-inducible transcript 4. Synonyms: Dig2, FLJ20500, HIF-1 responsive protein RTP801	10q22.1f	NM_019058.2	0.00438	0.65	Cell signaling. Involvement of RTP801 in amyloid β-peptide toxicity and pathogenesis AD [84]
HBB&	Hemoglobin, beta	11p15.4c	NM_000518.4	0.01216	0.66	Oxygen transporter in blood. Interactions between β-Amyloid and Hemoglobin has been shown [85]–[87].
HBA2&	Hemoglobin, alpha 2	16p13.3f	NM_000517.3	0.00606	0.69	Oxygen transporter in blood. Interactions between β-Amyloid and Hemoglobin has been shown [85]–[87].
IL7R	Interleukin 7 receptor	5p13.2c	XM_937367.1	0.0041	0.71	Cytokine receptor
HBA1&	Hemoglobin, alpha 1	16p13.3f	NM_000558.3	0.01676	0.72	Oxygen transporter in blood. Interactions between β-Amyloid and Hemoglobin has been shown ([85]–[87].
CD69	CD69 molecule	12p13.31a	NM_001781.1	0.00213	0.73	Cellular antigen
CLC	Charcot-Leyden crystal protein	19q13.2b	NM_001828.4	0.0237	0.75	Eosinophil lysophospholipase (galectin-10)
MYC	v-myc myelocytomatosis viral oncogene homolog (avian)	8q24.21b	NM_002467.3	0.01942	0.77	Myc proto-oncogene protein [88]
C4ORF18	Chromosome 4 open reading frame 18	4q32.1d	NM_016613.4	0.01343	0.77	Chromosome 19 open reading frame 43
CD36	CD36 molecule (thrombospondinreceptor)	7q21.11c	NM_000072.2	0.02719	0.79	CD36 antigen [89]
PRAGMIN	Homolog of rat pragma of Rnd2	8p23.1e	NM_001080826.1	0.01367	0.82	Tyrosine-protein kinase SgK223
**Up-regulated genes**						
MMP9	Matrix metallopeptidase 9	20q13.12b	NM_004994.2	0.01041	2.05	AD-presenilin pathway [90]–[92]
LOC441087	Hypothetical gene supported by AK125735	5q13.3c	NM_001013716.1	0.04332	1.86	No search result
KCNH6	Potassium voltage-gated channel,subfamily H (eag-related) member 6	17q23.3a	NM_030779.2	0.00243	1.86	Potassium voltage-gated channel Eag-related subfamily H member 6
UBN1	Ubinuclein 1	16p13.3b	NM_001079514.1	0.00259	1.84	Chromatin remodeling factor. A molecule that binds a nucleic acid. It can be an enzyme or a binding protein
CRISPLD2	Cysteine-rich secretory protein LCCLdomain containing 2	16q24.1a	NM_031476.2	0.00708	1.77	A specific protein substance that is produced to take part in various defense and immune responses of the body
LRG1	Leucine-rich alpha-2-glycoprotein 1	19p13.3d	NM_052972.2	0.00082	1.77	Cell adhesion molecules and their ligands A receptor that contains an immunoglobulin domain. It is often involved in the immune response [93]
IL1R2	Interleukin 1 receptor, type II	2q11.2e	NM_173343.1	0.00793	1.75	MAPK signaling pathway Type I cytokine receptor up-regulated in AD brain [94], [95]
USP10	Ubiquitin specific peptidase 10 (USP10)	16q24.1a	NM_005153.2	0.00046	1.70	Involvement in protein turnover and degradation is perturbed in AD [96], [97]
CMTM2	CKLF-like MARVEL transmembranedomain containing 2	16q21e	NM_144673.2	0.00386	1.66	A myelin protein found in the myelin sheath
PADI4	Peptidyl arginine deiminase, type IV	1p36.13d	NM_012387.1	0.00675	1.65	Catalyzes the fibrillogenesis of β-amyloid peptides [98]
GPR97	G protein-coupled receptor 97	16q13d	NM_170776.3	0.00608	1.64	Dysfunction of G protein-coupled receptor kinases in AD [97], [99]
RPSA	Ribosomal protein SA	3p22.1c	NM_002295.4	0.00492	1.63	A protein that comprises part of the ribosome
MME, Neprilysin&	Membrane metallo-endopeptidase	3q25.2c	NM_000902.3	8.0E-5	1.61	Neprilysin: amyloid-degrading peptidase [100], [101]
MOSC1	MOCO sulfurase C-terminal domain containing 1	1q41d	NM_022746.2	0.00401	1.61	Involvement in the stress process
VNN2	Vanin 2	6q23.2b	NM_004665.2	0.01584	1.60	Enzymes catalyzing the hydrolysis of a variety of bonds, such as esters, glycosides, or peptides
LOC642103	PREDICTED: Maltase-glucoamylase, intestinal		XM_936233.1	0.00039	1.57	No search results
TGM3	Transglutaminase 3	20p13d	NM_003245.2	0.00651	1.56	Acyltransferase: TGM3 and its regulator tazarotene-induced gene 3 localized to neuronal tau inclusions in tauopathies [102]
LILRA5	Leukocyte immunoglobulin-like receptor, subfamily A	19q13.42a	NM_021250.2	6.41E-6	1.55	Membrane-bound signaling molecule
NCF4	Neutrophil cytosolic factor 4	22q12.3d	NM_000631.3	0.00183	1.51	Not determined
SORL1&	Sortilin-related receptor, L (DLR class) A repeats-containing	11q24.1a	NM_003105.3	0.00314	1.47	Receptor activity: SORL1 is genetically associated with AD [103]–[104]
UBB	Ubiquitin B	17p11.2i	NM_018955.2	0.00948	1.37	Ubiquitin B: Its mutant form, polyubiquitin (UbB) +1, is implicated in neuronal cell death in AD [105], [106]

*KEGG, Kyoto Encyclopedia of Genes and Genomes (http://www.genome.jp/kegg/).

**The National Center for Biotechnology Information (http://www.ncbi.nlm.nih.gov).

& Genes selected for further analysis.

### Verifying microarray data with qRT-PCR

**Figure 3:**
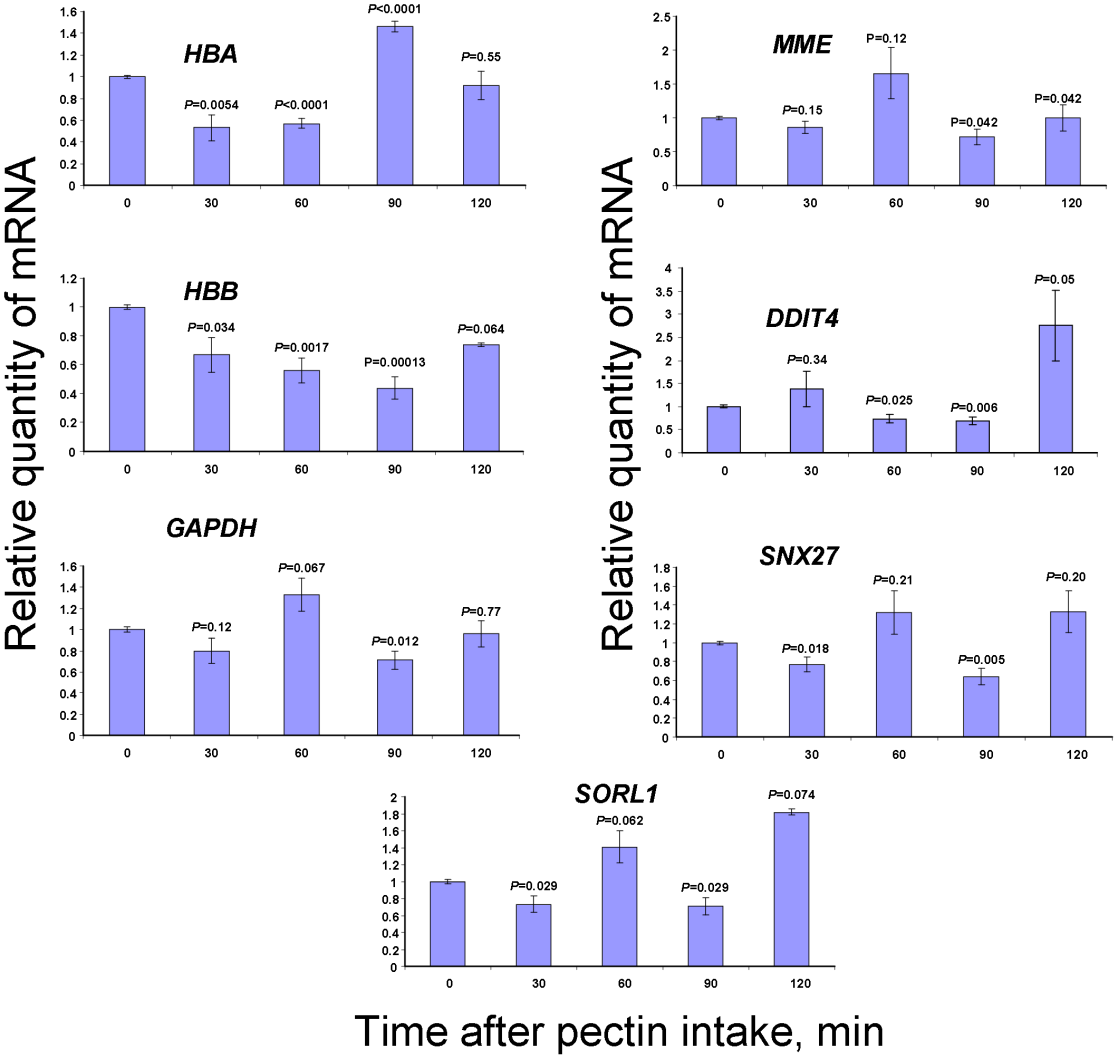
Pectin-generated MeOH affects gene expression in the WBC. The influence of citrus pectin intake on human blood leukocyte gene transcription as determined by qRT-PCR. The relative quantities of mRNA after pectin intake were normalized to the mRNA levels before pectin intake. Student’s *t*-test *P*-values were calculated by using triplicate blood samples from three volunteers.

Microarray data verification was conducted by using WBCs from volunteers after administering pectin or red wine. Oligonucleotide primers for the 7 genes related to oxygen transport (hemoglobin *HBB* and *HBA* genes), cell signaling (*DDIT4*), AD (*MME* and *SORL1*) and the earlier identified MRGs (*GAPDH* and *SNX27*) were used to validate the microarray data by qRT-PCR. Figure 3 shows the mRNA content changes of WBCs from volunteers after pectin intake. The *HBA*, *HBB*, *DDIT4, MME* and *SORL1* genes exhibited complicated dynamic changes in transcript abundance but generally confirmed the microarray assay data (Table 1). The *GAPDH* and *SNX27* genes missing in Table 1 also showed dynamic changes in their mRNA levels after pectin intake.

**Figure 4:**
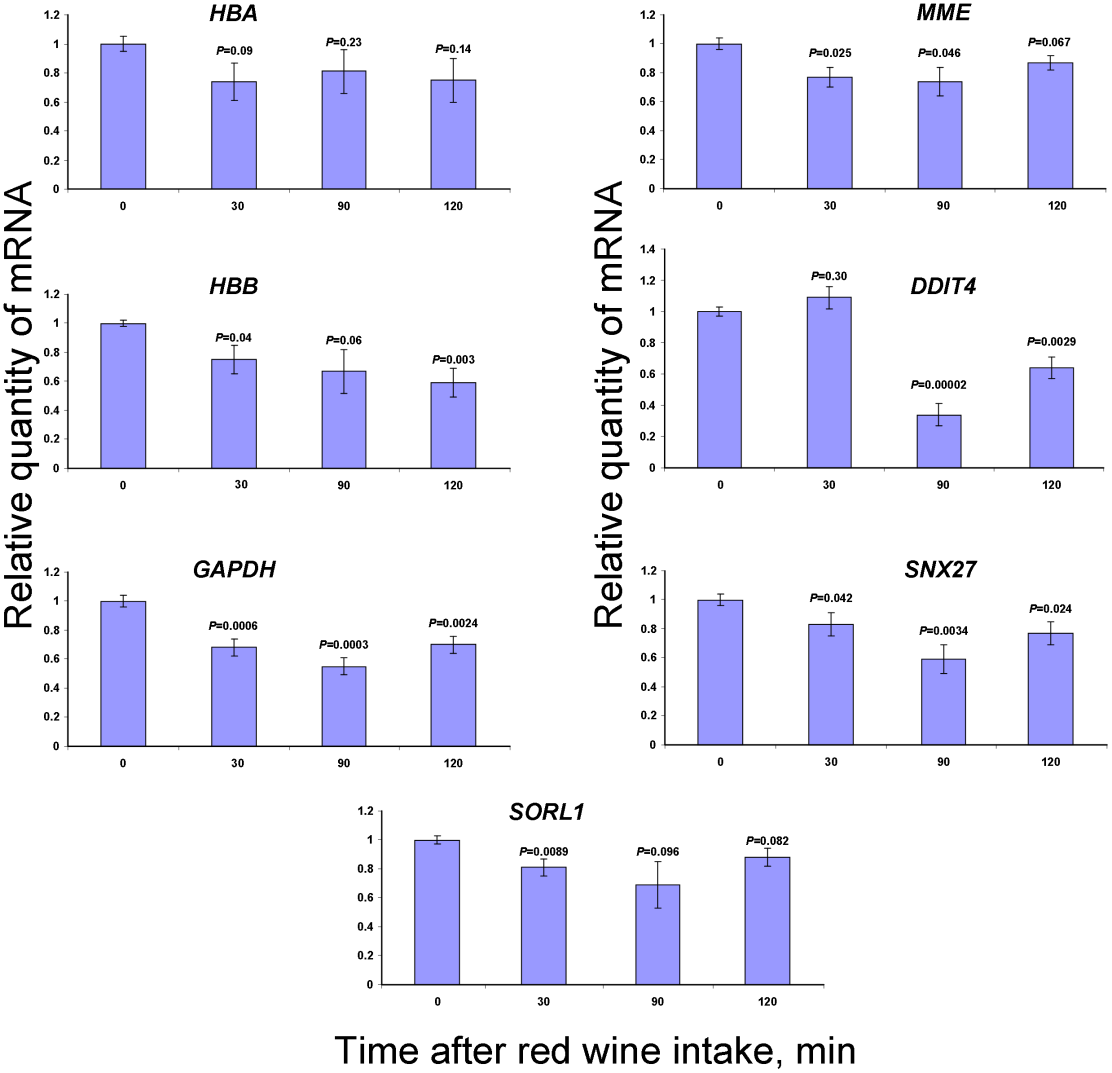
qRT-PCR analysis of WBC mRNA content after red wine intake. The relative mRNA quantities after red wine intake was normalized to the mRNA levels before red wine intake. Student’s *t*-test *P*-values were calculated by using triple bloods samples of three volunteers.

Next, we studied how MeOH concentration in the blood plasma correlates with the mRNAs accumulation in WBCs after red wine intake (Fig. 4). In contrast to our expectations, consuming EtOH-containing red wine does not alter the accumulation of mRNAs in the WBCs, which we obtained from volunteers after collecting MeOH-forming citrus pectin.

We concluded that pectin ingestion and red wine intake leads to a change in the MeOH and FA contents of human plasma, which is accompanied by a change in the mRNA abundance of genes involved in cell signaling.

### The brain genome-wide analysis of mice after inhalation of vapors of injured plants and of MeOH

**Figure 5:**
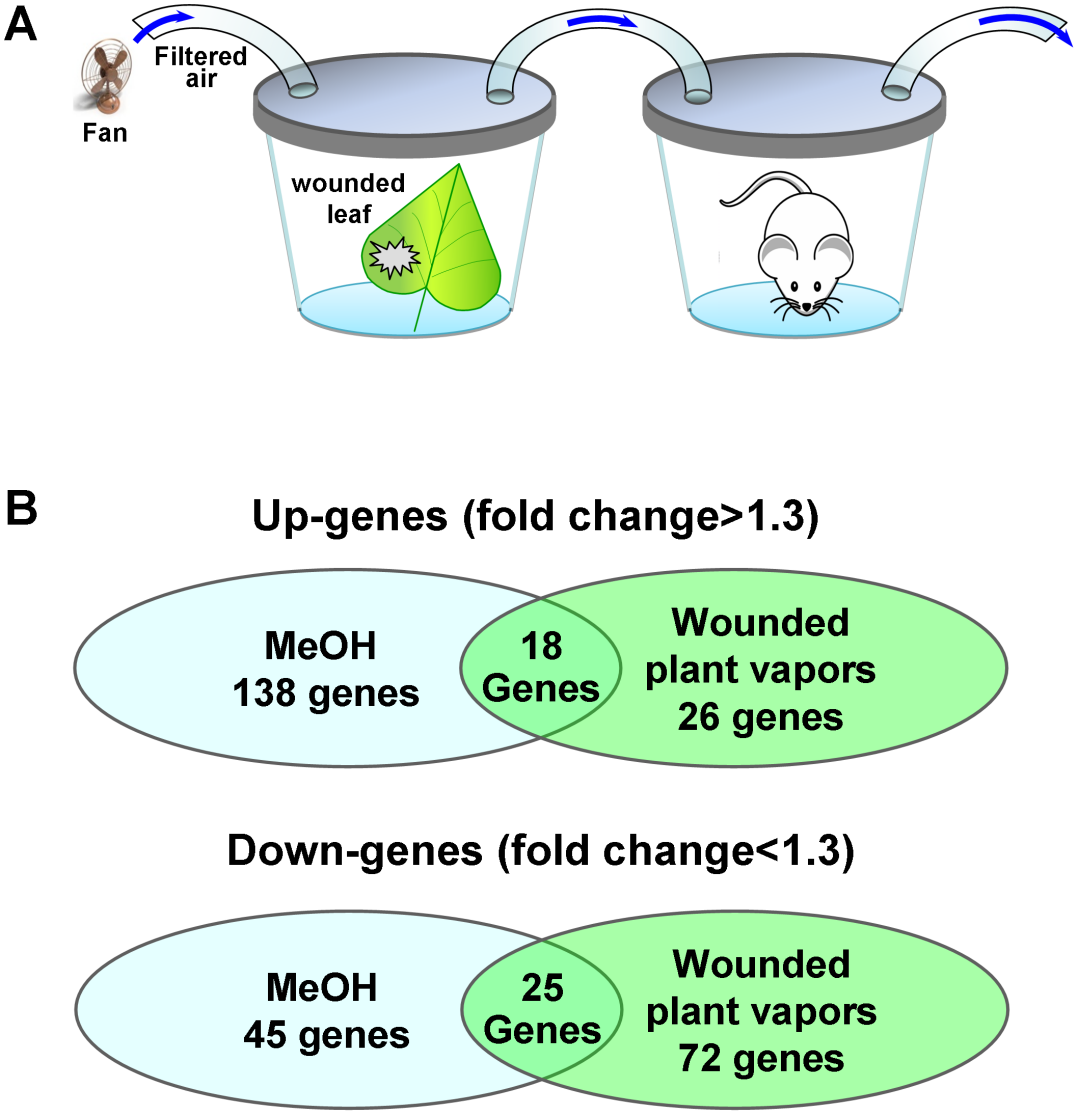
Microarray analysis of differentially regulated murine brain mRNAs after mouse inhalation of methanol and wounded leaf vapors. (A) Experimental set-up for the inhalation of vapors from wounded leaves by the mice. (B) Venn diagram of the genes that are differentially expressed after the inhalation of methanol and wounded leaf vapors compared with those after the inhalation of water vapor. The genes were analyzed using the J-Express gene expression analysis software. The number of genes commonly regulated is indicated in the intersection of the circles. All the genes (with an average fold-change ≥1.3-fold) included in this analysis showed significant changes in their expression compared with the control, with a *Q*-value <0.05.

**Figure 6:**
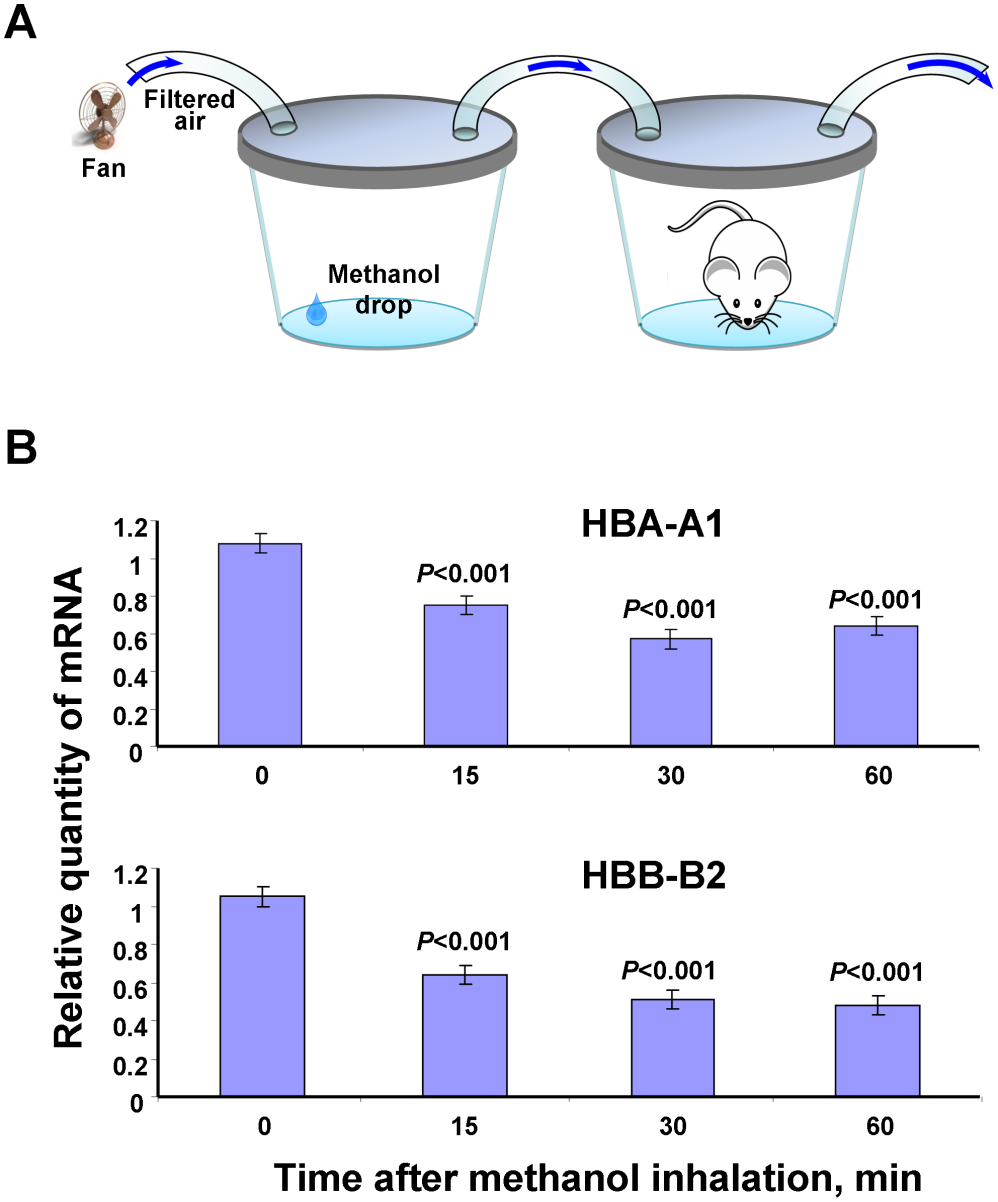
Verification of the microarray data for hemoglobin gene expression. (A) Experimental set-up for the MeOH inhalation by the mice. (B) qRT-PCR analysis of murine brain mRNAs for the hemoglobin genes after methanol inhalation. The data shown represent five independent experiments. ***P<0.001 (Student’s *t*-test).

The reduced mRNA synthesis of hemoglobin genes in WBCs may be a common reaction of cells to an increase in the plasma levels of MeOH. To test this hypothesis, we conducted a genome-wide analysis of the brain in mice after exposure to methanol vapor under conditions that were close to natural: the mice breathed the vapors from injured plants known to contain methanol [3] (Fig. 5A). RNA samples collected from the mouse brain after inhalation of the wounded plant leaf vapors or MeOH were used to generate cDNA. The brain RNA samples after the inhalation of methanol served as a positive control. The cDNA were analyzed using Illumina Whole-Genome 8 microarrays with probes for approximately 25,600 transcripts. We identified those transcripts that were expressed with *P*-values of <0.05. Figure 5B shows a Venn diagram of the genes that were differentially expressed after exposure to the vapors from wounded leaves and after methanol inhalation compared with those after the inhalation of water vapor, which served as a negative control. The number and names of the genes (Table S3 and S4) that were regulated by the inhalation of both the vapors from wounded leaves and from methanol are shown in the intersection of the circles (Fig. 5B). Notably, the transcriptional activity of the hemoglobin genes *mHBA-A1* and *mHBB-B2* (Table S3), which appear to be the genes most sensitive to methanol, is reduced, as in the case of human WBCs (Table 1). The presence of hemoglobin mRNAs in the brain cells was not a result of contamination with red blood cells because the levels of erythrocyte-specific marker genes, including *GYPA*, *EBP4*, *SPTA* and *ALAS2*, were not significantly expressed. Verification of these microarray data by qRT-PCR showed a dynamic diminution in the transcript abundance of the hemoglobin genes during the first hour after MeOH inhalation (Fig. 6).

We concluded that the inhalation of MeOH vapors led to a change in the mRNA abundance of the *mHBA-A1* and *mHBB-B2* genes in mouse brain.

## Discussion

To assess whether methanol is a friend or foe to humans, we must consider the favorable role of fruits and raw vegetables in human health. Vegetables in the diet are the primary source of exogenous MeOH for a healthy person [3], [109]. The role of MeOH-generating pectin in atherosclerosis and cancer prophylaxis is well-known [110]–[114]. Pectin is believed to affect detoxifying enzymes, stimulate the immune system, modulate cholesterol synthesis and act as an antibacterial, antioxidant or neuroprotective agent [115]. There is also increasing evidence to suggest the regular consumption of fruits and vegetables may play an important role in preventing or delaying the onset of dementia, age-associated cognitive decline and AD [82], [116]–[120].

However, in considering the function of methanol, it is important to estimate the production of endogenous methanol in humans and its toxic metabolites. To study the role of vegetable diet in endogenous methanol generation, we used citrus pectin, which is a dietary supplement. We previously showed that citrus pectin efficiently generates MeOH in the mouse gastrointestinal tract [3]. In this study, we confirmed the ability of citrus pectin to generate MeOH in the human gastrointestinal tract. Two hours after citrus pectin intake (6.0 g), the MeOH content increased by almost two times in volunteer plasma from three independent experiments (Fig. 1). We tried to estimate the level of endogenous MeOH generation by considering the probable removal of MeOH through pulmonary and renal excretion. To estimate the influence of renal clearance on the plasma MeOH balance, we used urine formation parameters for a healthy average ≈70-kg person who consumes alcohol at approximately 60 ml/h, or 0.86 ml/h/kg [22], [121]. Renal MeOH clearance in the average volunteer was measured as 1.0 ml/min or 15 ml per 15 min; it is as if 15 ml of blood was totally cleared of MeOH every 15 minutes. Assuming the average man had a body water content of 60%, and the blood weight accounted for 7% of the body weight we believe that a clearance of 60 ml of blood in 1 hour has little effect on the total MeOH content of the blood. With regards to the pulmonary excretion of MeOH, we used estimates [122] indicating for each minute, 5.6 ml of blood is hypothetically and totally cleared of MeOH, i.e., the effect of pulmonary clearance of plasma MeOH is also negligible. Ignoring the low contribution of renal and pulmonary clearance, we estimated the approximate level of MeOH formation as 116 mg/h by evaluating the endogenous sources of a 70-kg person, i.e., the lower level of endogenous MeOH production is at least ≈1.66 mg/kg/h. Because it seems unlikely that EtOH is able to deplete all of the liver ADH, we should be allowing for a higher level of MeOH production. If we use the lower bound for the formation rate of endogenous MeOH at 1.66 mg/kg/h, then a 70-kg person can form at least 116 mg of MeOH in 1 hour, which is comparable with ingesting 274 ml of red wine.

Elucidating the biological significance of physiological MeOH can help to locate biological and gene targets in WBCs. The potential advantage of blood-based biomarkers is obvious; collecting blood is easier than almost any other body fluid, and blood-based tests lend themselves to high-throughput and inexpensive measurements [123]. There is relatively little evidence available about a transcript signature in the WBCs of AD that might act as a biomarker [124]–[127]. However, the digestion of food by itself has an effect on WBC transcriptional activity [128]–[130]. Thus, to minimize the impact of food intake, we had to provide a small amount of pectin, which leads to MeOH formation in the digestive tract of volunteers.

The identification of human genes, the transcriptional activity of which varies with the blood levels of MeOH, would lie between three possible functions of MeOH in humans as follows: (a) MeOH, a poisonous biochemical waste product, (b) MeOH, a signaling molecule that regulates the activity of human life processes, and (c) a combination of the two above-mentioned mechanisms, that is, MeOH, a Janus-like substance similar to carbon dioxide that is released from the body during respiration, but without which the brain respiration centers cannot be activated [131].

Our microarray analysis of volunteer WBCs after pectin intake showed the various responses of differentially regulated mRNAs (Table 1). Of the 32 genes selected for analysis, 30 were also found to be annotated through the DAVID database service (http://david.abcc.ncifcrf.gov). As shown in the histogram (Fig. S8), half the genes encoded glycoproteins, and their expression is altered with increased blood levels of MeOH. Most of the present genes exhibit increased expression in response to increasing amounts of MeOH (8/15, 53%), and the others are down-regulated (7/15, 47%). Hemoglobin genes exhibit the most pronounced decrease in mRNA synthesis in WBC. This effect is not an artifact and cannot be explained by erythroblast contamination of the WBCs. This finding is supported by our failure to detect the presence of specific erythrocyte gene markers, including *GYPA*, *EBP4*, *SPTA* and *ALAS2*. Moreover, this reaction is characteristic of cells in general and is not blood-related. Our experiments on animals have shown that the brains of mice exposed to methanol respond by decreasing the levels of hemoglobin gene expression (Fig. 6).

Interestingly, of the 30 proteins analyzed, 8 proteins are receptors, all of which are located in the plasma membrane; 5 of them are up-regulated (68%), and the remaining three are down-regulated (32%). Perhaps this regulation is explained by the increased amount of MeOH in the intercellular space and its impact on these receptors. According to the PANTHER database (http://www.pantherdb.org), 9 of the 32 proteins participated in cell-cell communication processes (Fig. S9). All these findings favor the idea that MeOH concentration changes effect the expression of genes of intercellular communication in WBC. We used the STRING database (http://string-db.org) to analyze possible links between the products of selected genes. The interactions shown in the image (Fig. S10), which was built with a medium confidence parameter (≥0.400), predicted interactions via CD36 between MMP9 and hemoglobins, and among MMP9 and MYC and NCF4. According to KEGG (http://www.genome.jp/kegg/pathway.html), NCF4 and MMP9 are involved in lymphocyte migration, and their interaction is mediated by ROS, such as hydrogen peroxide. In the case of increasing MeOH concentrations in the blood, the expression of *NCF4* and *MMP9* genes is likely to rise, suggesting their possible positive interaction as induced by MeOH. The relation between MMP9 and MYC is also interesting, as predicted from the KEGG database and the NCI-Nature Pathways Interaction Database. MYC is a known oncogene, and it affects the transcription of a huge number of genes in combination with MAX (Myc-associated factor X, activating complex) and/or MIZ-1 (Myc-interacting zinc finger protein 1, inhibitory complex). As shown in our microarray expression analysis, given an increased amount of MeOH in the blood, the *MYC* expression in WBC is reduced, whereas *MMP9* increased. Perhaps MYC complexes with other proteins and exerts an inhibitory effect on *MMP9* expression, whereas the changing amount of MeOH in the blood reduces the level of *MYC* expression directly or indirectly. Thus, the reduced number of inhibitory complexes with MYC could lead to an increase in the *MMP9* expression. In addition to interaction with *MMP9*, MYC was experimentally demonstrated to directly interact with UBB [132] (Fig. S10).

Thus, our analysis allows us to cede to the third hypothesis, which includes not only the inevitable involvement of MeOH-to-FA toxic metabolite formation but also the participation of MeOH in the regulation of gene transcriptional activity.

## Materials and Methods

### Volunteer experiments

Eight healthy male and female volunteers between 21 and 67 years of age and within 15% of the normal weight range for their age, height, and frame size were selected for this study. A complete medical history and medical examination, including chest X-ray, electrocardiogram, hematology screening, serum chemistry, and urine analysis, were conducted to assess the health statuses of the volunteers. Exclusion criteria included smoking, obesity, a family history of chronic diseases, any use of medication, heavy physical exercise (10 h/week), pregnancy, and breastfeeding. No subjects had evidence of acute or chronic disease, and all subjects had normal values for the various tests. These studies were approved by the Human Subjects Committee at the N. N. Blokhin National Cancer Research Center, Moscow, Russia (Extract from the Bioethics Commission meeting number 2, on September 20, 2011). Informed consent was obtained from all subjects with written approved consent forms.

#### Citrus pectin intake

The subjects fasted for 12 hours prior to beginning each experiment and evaluation session. Each volunteer swallowed capsules with PME containing citrus pectin (6 g) (Nittary Pharmaceuticals, VitaLine, Inc., USA). After 30, 60, 90 and 120 min, blood and saliva samples were obtained and analyzed for methanol and formaldehyde content by GC and HPLC, respectively.

#### Red wine administration

The subjects fasted for 12 hours prior to beginning the experiment and evaluation session. Each volunteer drank 150 ml of red wine (13.731% ethanol with 0.0424% methanol content). After 15, 30, 60, 90 and 120 min, blood and saliva samples were collected and analyzed for methanol, ethanol and formaldehyde content by GC and HPLC, respectively.

#### Alcohol administration

The subjects fasted for 12 hours prior to beginning the experiment and evaluation session. Each volunteer drank 50–80 ml of 40% alcohol (1 ml per kg of body weight). After 15, 30, 60, 90 and 120 min, blood and saliva samples were collected and analyzed for methanol, ethanol and formaldehyde content by GC and HPLC, respectively.

### Human WBC preparation

The blood samples were homogenized with 10 ml of modified Hanks balanced salt solution (Sigma-Aldrich). WBCs were prepared by Ficoll density-gradient centrifugation (Ficoll-Paque PLUS; Amersham) at 300 g for 30 min at 20°C. The ring of high-density WBCs was isolated and washed twice in 50 ml of Hanks buffer. After determining cell survival by trypan-blue exclusion test, the WBC concentration was normalized to 10^6^ cells/ml in Hanks buffer. WBC populations were evaluated by microscopic observation after May-Grunwald-Giemsa staining. Cells were centrifuged for 6 min at 700 g (4°C), and then 100 µl of 1× PBS was immediately added to the pellet. Total RNA was isolated from WBCs with TriReagent (MRC, USA) according to the manufacturer’s protocol. For the methanol measurements, blood samples without anticoagulant were incubated at 4°C for 2 h to allow cell sedimentation to occur, and an equal volume of 10% trichloroacetic acid was then added to the plasma aliquot. The mixture was incubated for 20 min on ice and then centrifuged for 10 min at 16 000 g. Finally, the supernatant was analyzed for methanol content by GC.

### Animal experiments

Experiments were performed on male BALB/c mice. The animals had unlimited access to food and water and were kept in cages with a temperature controlled environment (20±1°C) with the lights on from 9 AM to 9 PM. The care of experimental animals was performed in strict accordance with the guidelines of the ‘‘Euroguide on the accommodation and care of animals used for experimental and other scientific purposes’’ (FELASA, 2007). All experimental protocols were approved by the Animal Ethics Committees of the A. N. Belozersky Institute of Physico-Chemical Biology, Moscow State University, Moscow, Russia (Protocol Registration number 2/12 of 6^th^ February 2012). Euthanasia was performed using carbon dioxide in accordance with the 2000 Report of the AVMA Panel on Euthanasia, and all efforts were made to minimize animal suffering. For all surgical procedures, rats were anesthetized by intraperitoneal injection of 300 mg/kg (12%) chloral hydrate. Additionally, to ensure proper pain relief in the preoperative and postoperative periods, we used repeated topical application of a long-acting local anesthetic bupivacaine ointment. Moribund animals, animals obviously in pain, or animals showing signs of severe and enduring distress were humanely killed. Criteria for making the decision to kill moribund or severely suffering animals, and guidance on the recognition of predictable or impending death, were performed in accordance with the Guidelines for Endpoints in Animal Study Proposals (DHHS NIH Office of Animal Care and Use).

### Methanol and ethanol measurements by gas chromatography

The methanol and ethanol contents were determined by GC on a capillary FFAP column (50 m×0.32 mm; Varian Inc., Lake Forest, CA, USA) in a Kristall 2000 gas chromatograph (Eridan, Russia). Liquid samples were measured under the following operating conditions: carrier gas, nitrogen; nitrogen flow, 30 ml/min; air flow, 400 ml/min; hydrogen flow, 40 ml/min; injected volume, 1 µl; injector temperature, 160°C; column temperature, 75°C, column temperature increased at 15°C/min to 150°C; retention time, 6.5 min (methanol) or 6.43 min (ethanol); and flame ionization detector temperature, 240°C.

### Formaldehyde measurements by HPLC

The Dionex Ultimate 3000 HPLC system was used, and it consisted of a four-gradient pump, a degasser mobile phase, an automatic injector (autosampler) combined with a column thermostat and a spectrophotometric detector with a variable wavelength detector (with simultaneous detection according to 4 different wavelengths). The selected chromatographic column was the Synergi Hydro-RP, 250 mm×4.6, with a sorbent grain diameter of 4 microns (porosity 80 Å) and a pre-column Security Guard (C18 cartridge, 3 mm diameter) (manufactured by Phenomenex, USA). The stationary phase was silica, and it was grafted with polar groups and end-capped by C18. The mobile phase was a mixture of deionized water and acetonitrile (HPLC grade) in a 50/50 ratio by volume. The mobile phase flow rate was 1 ml/min, the column temperature control was set to 30°C, and the sample injection volume was 20 µl of a pre-washing injector mixture of acetonitrile and water (50/50) plus the sample. The total analysis time was 20 minutes. The detection was conducted on a spectrophotometer flow cell by reading the absorbance at 360 nm (near-UV). This assay was based on the interaction of formaldehyde with an excess of 2,4-dinitrophenylhydrazine in an acidic medium to form the corresponding colored hydrazone product, which was then separated from the remaining components of the solution by chromatography. To obtain the reagent solution, 100 µl of 85% phosphoric acid was added to 20 ml of pure acetonitrile, and then 20 mg of 2,4-dinitrophenylhydrazone hydrochloride was added. To prepare the blank solution, 0.5 ml of deionized water was added to 0.5 ml of reagent solution and stirred. The blank solution accounts for any minor impurities in the 2,4-dinitrophenylhydrazone of the formaldehyde in the reagent that formed during storage (for example, from exposing the formaldehyde to the atmosphere). For the sample measurement, 450 µl of deionized water was added to 50 µl of the test sample and 0.5 ml of reagent solution and stirred. The reaction proceeded at room temperature (22–24°C) for 20 min, after which the solution was injected into the chromatograph. For quantitative analysis, the actual calibration dependence on formaldehyde was determined by using a series of standard solutions. The equation for gradient dependence was S (peak area, arbitrary units) = 14.36×c (formaldehyde concentration in mg/l). R2 = 1, which accounted for the blank experiment. The formula for calculating the formaldehyde content of the samples was C (mg/L) = (S_x_–S_blank_)/14.36×20, where 14.36 is the calibration coefficient and 20 is the dilution factor.

### Human WBC microarrays

RNA was extracted from WBCs with an RNeasy Mini Kit (Qiagen) according to the manufacturer’s instructions. The Illumina BeadArray with a single-color array (Illumina, San Diego, CA, USA) was used as a microarray platform. For the Illumina BeadArray assay, cRNA was synthesized with an Illumina RNA Amplification Kit (Ambion, Austin, TX, USA) according to the manufacturer’s instructions. In brief, 400 ng of total RNA from WBCs was reverse-transcribed to synthesize first- and second-strand cDNA, which was purified with spin columns and then transcribed *in vitro* into biotin-labeled cRNA. A 750 ng quantity of biotin-labeled cDNA was hybridized to each Illumina Human NT-12 v.4 BeadChip array (Illumina) at 55°C for 18 h. The hybridized BeadChip was washed and labeled with streptavidin-Cy3 (GE Healthcare) and then scanned with the Illumina BeadStation 500 System (Illumina). The scanned image was imported into BeadStudio software (Illumina) for analysis. Approximately 45,000 transcripts representing twelve whole-genome samples can be analyzed on a single BeadChip. The correlation coefficient for identical RNAs was 0.993–0.998 (r2) in the present study. Data analysis was performed with GenomeStudio software (Illumina) and J-Express 2012. The microarray data has been deposited to GEO database with accession number GSE58350.

### Mouse brain microarrays

BALB/c mice were randomly divided into groups of five mice. RNA samples collected from the brain after the mouse inhalation of wounded plant leaves or MeOH vapors were used to generate cDNA. The mice were placed in a five-liter plastic container, which received air that was blown down (150 liters/hour) from the evaporator, a 250 ml flask with wounded leaves rubbed with Celite from the *Brassica rapa pekinensis* (approximately 1 g), or cotton wool soaked with 200 µl of methanol or water (control). After one hour, the brain samples were collected after decapitation. The RNA concentrations were determined using a Nanodrop ND-1000 spectrophotometer (Isogen Life Sciences, Netherlands). Whole brain homogenates from biological replicates were subjected to RNA isolation using TRIzol (Invitrogen, USA), according to the manufacturer’s instructions. Following isolation, total RNA was purified and concentrated using the RNeasy MinElute Kit (QIAGEN, Hilden, Germany). Total RNA (400 ng) was prepared for microarray using the Illumina TotalPrep RNA Amplification Kit (Ambion, USA). The brain transcriptome was assessed using Illumina MouseRef-8 BeadChip microarrays, which contain 25,600 specific oligonucleotide probes. Arrays were scanned using the Illumina BeadArray Reader and BeadScan software. Data were analyzed using GenomeStudio v.2012 (Illumina, USA) with normalization by Cubic Spline and differential expression analysis using the Illumina Custom algorithm. This analysis generated a list of probes with significant (*P*<0.05) differences in signal intensity between treated and control mice. Probe annotations from the microarray manifest file were updated using the SOURCE database (http://smd.stanford.edu/cgi-bin/source/sourceSearch) with the listed NCBI transcript accession numbers as the search terms. In cases where the accession number was no longer listed in the database, annotations were updated by aligning the probe sequence against the mouse transcriptome using BLAST (http://blast.ncbi.nlm.nih.gov/Blast.cgi). Genes were analyzed using the J-Express gene expression analysis software, SAM (Significance Analysis of Microarrays) tool. The microarray data has been deposited to GEO database with accession number GSE58303.

### qRT-PCR Analysis of Transcript Concentrations

The RNA concentrations were determined by using a Nanodrop ND-1000 spectrophotometer (Isogen Life Sciences). All RNA samples had a 260∶280 absorbance ratio between 1.9 and 2.1. cDNA was obtained by annealing 2 µg of denatured total RNA with 0.1 µg of random hexamers and 0.1 µg of Oligo-dT. The mixture was then incubated with 200 units of Superscript II reverse transcriptase (Invitrogen, USA) for 50 min at 43°C. qRT-PCR was performed by using the iCycler iQ real-time PCR detection system (Bio-Rad, Hercules, CA, USA). EVA Green master mix (Syntol, Russia) was used to detect the target genes according to the manufacturer's instructions. The thermal profile for EVA Green qRT-PCR included an initial heat-denaturing step at 95°C for 3 min followed by 45 cycles of the following: 95°C for 15 s, an annealing step (Table S5) for 30 sec and 72°C for 30 sec, coupled with fluorescence measurements. Following amplification, the melting curves of the PCR products were monitored from 55–95°C to determine the amplification specificity. Each sample was run in triplicate, with a no-template control added to each run.

## Supporting Information

Figure S1 **The methanol change dynamic in blood plasma and saliva after pectin intake.** The standard error bars are indicated. ***P<0.001 (Student’s *t*-test); n.s., not significantly different.
(TIF)

Figure S2 **A chromatogram picture showing the GC analysis results for ethanol contents in the red wine used for the human volunteer experiments.**
(TIF)

Figure S3 **A chromatogram picture showing the GC analysis results for the methanol content of the red wine used in human volunteer experiments.**
(TIF)

Figure S4 **Methanol change dynamic in blood plasma and saliva after red wine intake.** The standard error bars are indicated. ***P<0.001 (Student’s *t*-test).
(TIF)

Figure S5 **A chromatogram picture showing the GC analysis results for the methanol content of the 40% ethanol used in human volunteer experiments.**
(TIF)

Figure S6 **Methanol change dynamics in the blood plasma of male and female volunteers after administering 40% ethanol.** The standard error bars are indicated. *P<0.05; **P<0.01; ***P<0.001 (Student’s *t*-test); n.s., not significantly different.
(TIF)

Figure S7 **Cluster diagram showing the expression of 100 significantly differently expressed WBC genes before and after pectin intake.** The genes are organized by hierarchical clustering based on the overall similarity in expression patterns. Red represents a relative expression greater than the median expression level across all samples, and blue represents an expression level lower than the median. White indicates intermediate expression.
(TIF)

Figure S8 **A biological processes diagram for differentially expressed genes after pectin intake.** The genes were analyzed by using the DAVID database (http://david.abcc.ncifcrf.gov).
(TIF)

Figure S9 **A biological processes diagram for differentially expressed genes after pectin intake.** The genes were analyzed with the PANTHER database (http://www.pantherdb.org).
(TIF)

Figure S10 **A map of MeOH-sensitive proteins from the STRING database (** http://string-db.org **).** Colored lines indicate different sources of evidence for each interaction as follows: yellow line, both proteins are collectively found in PubMed; purple line, the interaction of the two proteins has been demonstrated experimentally; and blue line, proteins are often mentioned together in gene and protein databases.
(TIF)

Table S1 **The list of human up-regulated genes of volunteers after pectin intake.**
(DOC)

Table S2 **The list of human down-regulated genes of volunteers after pectin intake.**
(DOC)

Table S3 **The list of down-regulated genes in intersection of the Venn diagram circles presented in** **Figure 5B** **.**
(DOC)

Table S4 **The list of up-regulated genes in intersection of the Venn diagram circles presented in** **Figure 5B** **.**
(DOC)

Table S5 **Oligonucleotides used for qPCR.**
(DOC)
