# Supplementary figures and images for: Dietary Methanol Regulates Human Gene Activity
Source: PLoS One. 2014 Jul 17;9(7):e102837. doi: 10.1371/journal.pone.0102837 (PMC4102594; doi:10.1371/journal.pone.0102837)

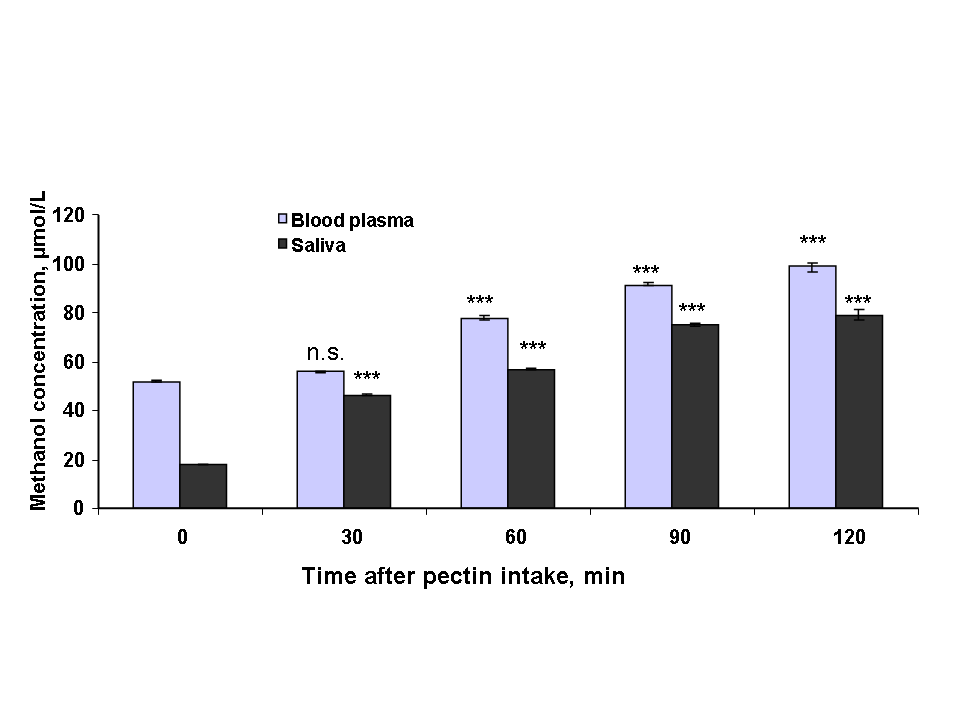

Supplement: Figure S1 — The methanol change dynamic in blood plasma and saliva after pectin intake. The standard error bars are indicated. ***P<0.001 (Student’s t-test); n.s., not significantly different. (TIF) [file pone.0102837.s001.tif]

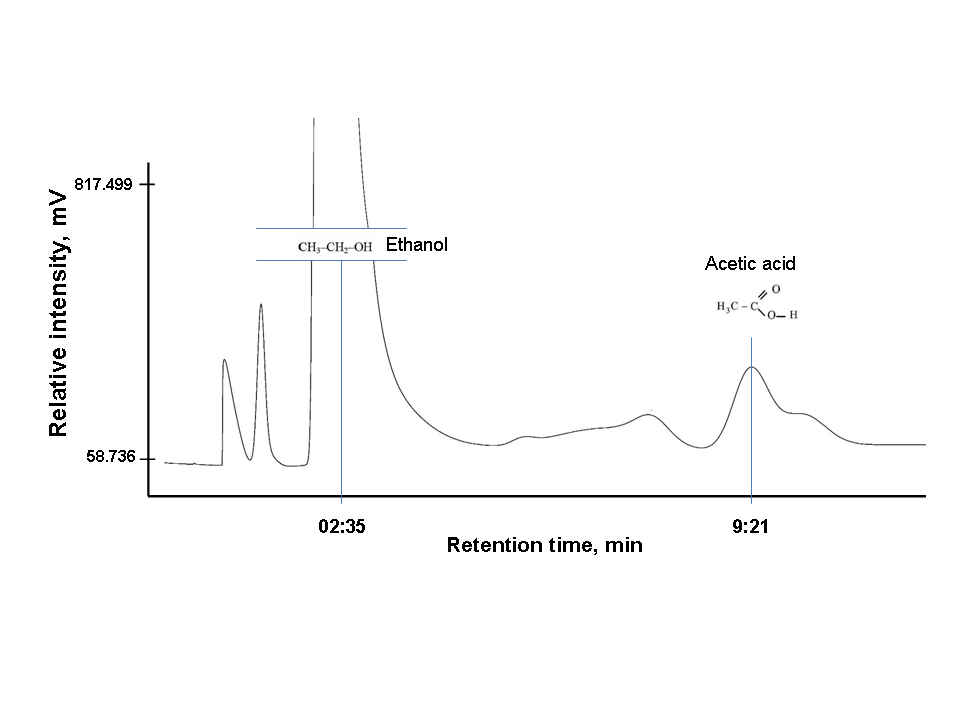

Supplement: Figure S2 — A chromatogram picture showing the GC analysis results for ethanol contents in the red wine used for the human volunteer experiments. (TIF) [file pone.0102837.s002.tif]

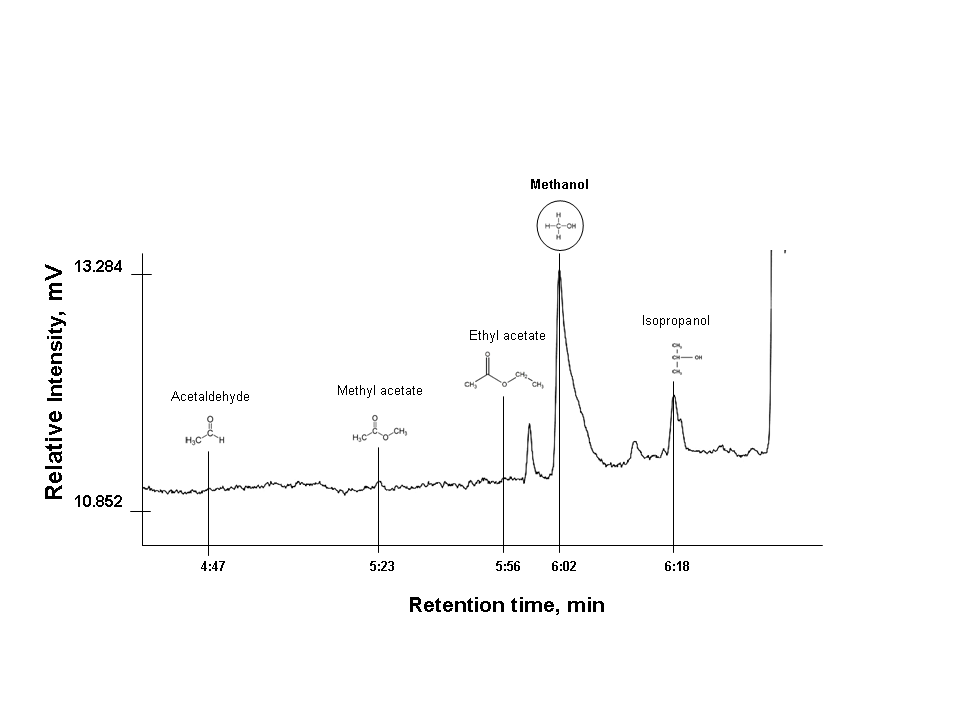

Supplement: Figure S3 — A chromatogram picture showing the GC analysis results for the methanol content of the red wine used in human volunteer experiments. (TIF) [file pone.0102837.s003.tif]

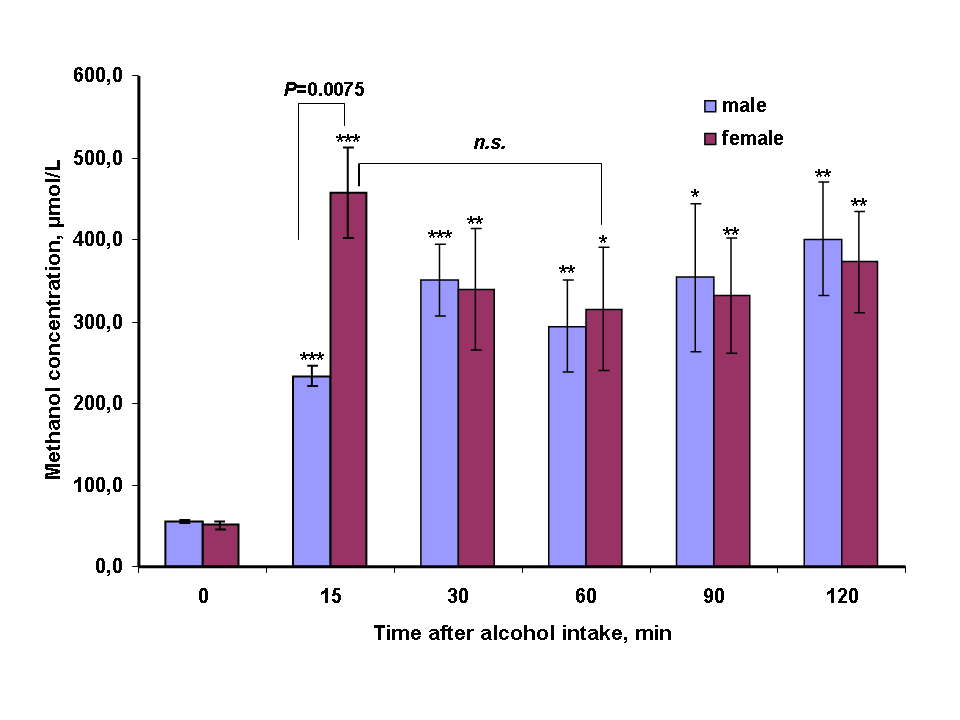

Supplement: Figure S6 — Methanol change dynamics in the blood plasma of male and female volunteers after administering 40% ethanol. The standard error bars are indicated. *P<0.05; **P<0.01; ***P<0.001 (Student’s t-test); n.s., not significantly different. (TIF) [file pone.0102837.s006.tif]

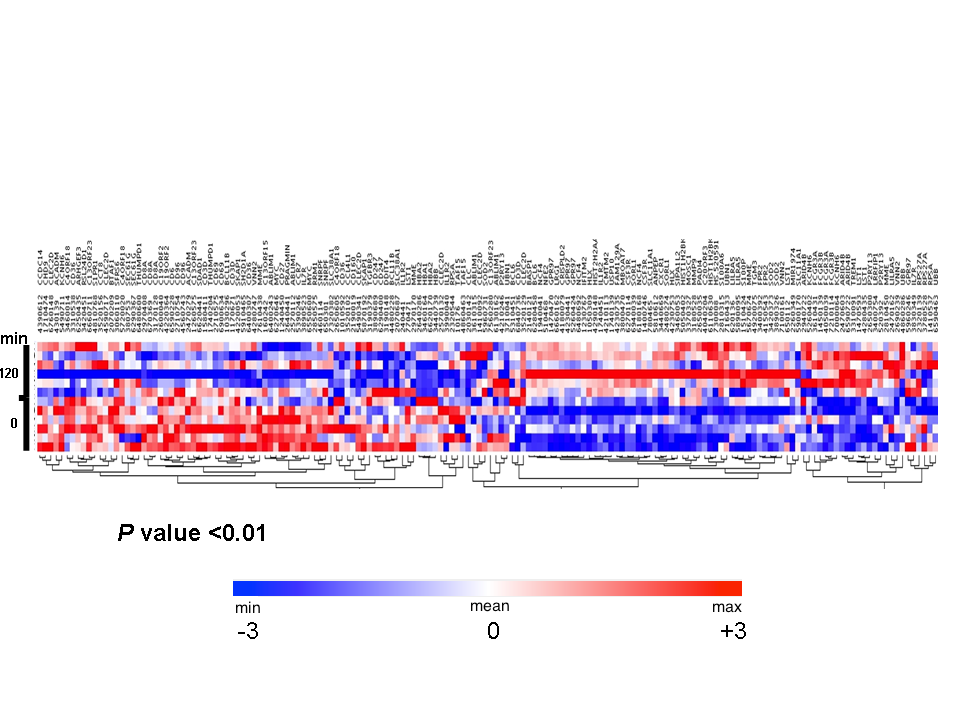

Supplement: Figure S7 — Cluster diagram showing the expression of 100 significantly differently expressed WBC genes before and after pectin intake. The genes are organized by hierarchical clustering based on the overall similarity in expression patterns. Red represents a relative expression greater than the median expression level across all samples, and blue represents an expression level lower than the median. White indicates intermediate expression. (TIF) [file pone.0102837.s007.tif]

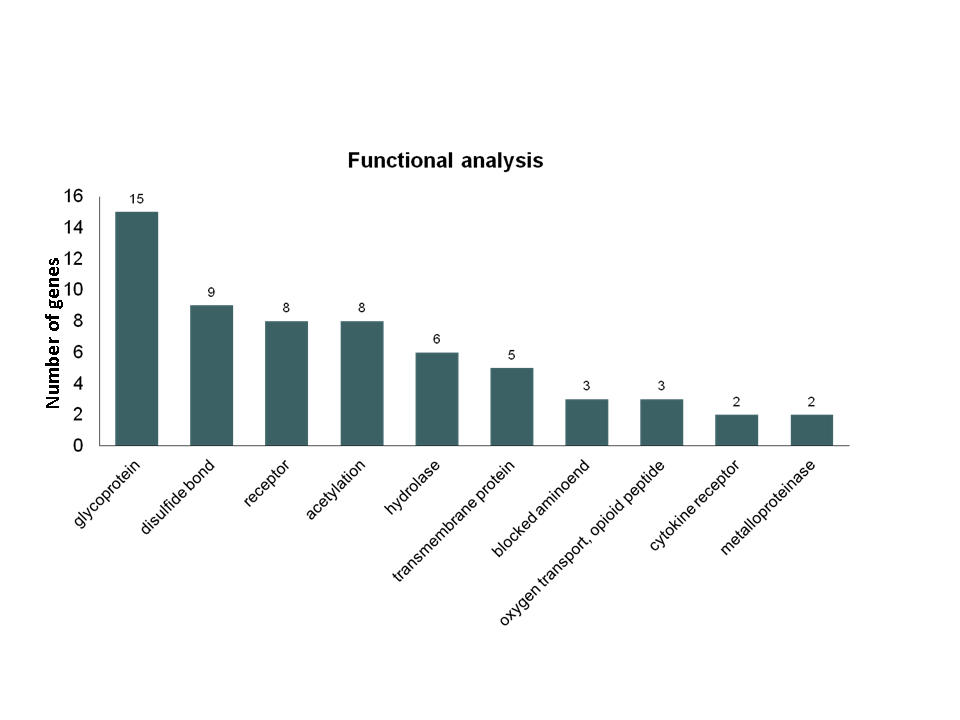

Supplement: Figure S8 — A biological processes diagram for differentially expressed genes after pectin intake. The genes were analyzed by using the DAVID database (http://david.abcc.ncifcrf.gov). (TIF) [file pone.0102837.s008.tif]

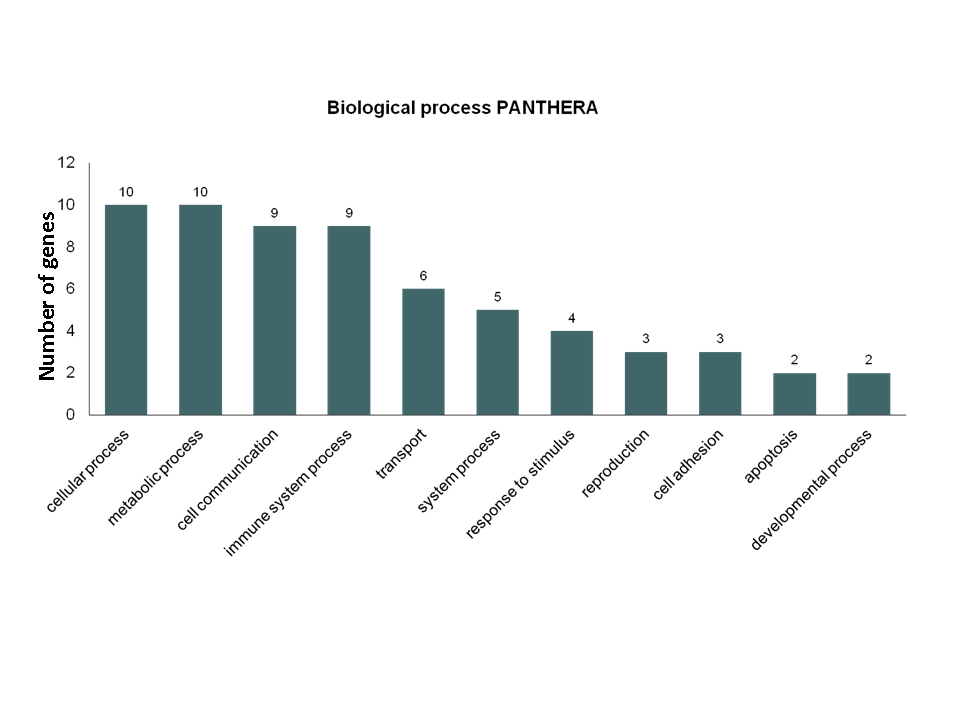

Supplement: Figure S9 — A biological processes diagram for differentially expressed genes after pectin intake. The genes were analyzed with the PANTHER database (http://www.pantherdb.org). (TIF) [file pone.0102837.s009.tif]

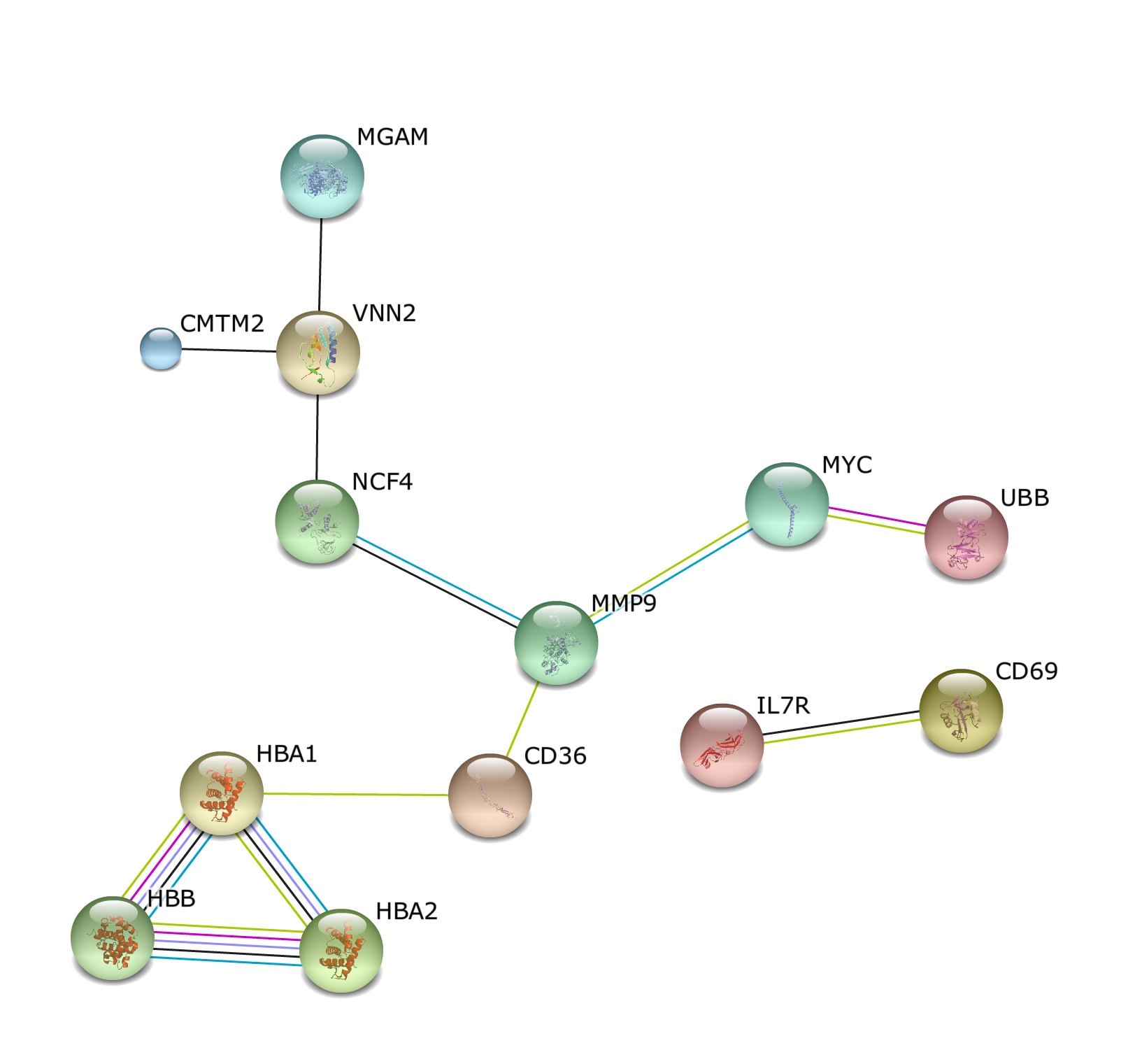

Supplement: Figure S10 — A map of MeOH-sensitive proteins from the STRING database ( http://string-db.org ). Colored lines indicate different sources of evidence for each interaction as follows: yellow line, both proteins are collectively found in PubMed; purple line, the interaction of the two proteins has been demonstrated experimentally; and blue line, proteins are often mentioned together in gene and protein databases. (TIF) [file pone.0102837.s010.tif]
