# Supplementary material for: Dietary Methanol Regulates Human Gene Activity
Source: PLoS One. 2014 Jul 17;9(7):e102837. doi: 10.1371/journal.pone.0102837 (PMC4102594; doi:10.1371/journal.pone.0102837)
Supplement: Table S1 — The list of human up-regulated genes of volunteers after pectin intake. (DOC) [file pone.0102837.s011.doc]

| TargetID | Accession | Cytoband | q-value | Fold Change |
| --- | --- | --- | --- | --- |
| MMP9 | NM_004994.2 | 20q13.12b | 0.01041 | 2.05 |
| KCNH6 | NM_030779.2 | 17q23.3a | 0.00243 | 1.86 |
| LOC441087 | NM_001013716.1 | 5q13.3c | 0.04332 | 1.86 |
| UBN1 | NM_001079514.1 | 16p13.3b | 0.00259 | 1.84 |
| CRISPLD2 | NM_031476.2 | 16q24.1a | 0.00708 | 1.77 |
| LRG1 | NM_052972.2 | 19p13.3d | 0.00082 | 1.77 |
| IL1R2 | NM_173343.1 | 2q11.2e | 0.00793 | 1.75 |
| USP10 | NM_005153.2 | 16q24.1a | 0.00046 | 1.70 |
| CMTM2 | NM_144673.2 | 16q21e | 0.00386 | 1.66 |
| PADI4 | NM_012387.1 | 1p36.13d | 0.00675 | 1.65 |
| GPR97 | NM_170776.3 | 16q13d | 0.00608 | 1.64 |
| RPSA | NM_002295.4 | 3p22.1c | 0.00492 | 1.63 |
| MME | NM_000902.3 | 3q25.2c | 0 | 1.61 |
| MOSC1 | NM_022746.2 | 1q41d | 0.00401 | 1.61 |
| VNN2 | NM_004665.2 | 6q23.2b | 0.01584 | 1.60 |
| LOC642103 | XM_936233.1 |  | 0.00039 | 1.57 |
| GPR1 | NM_005279.2 | 2q33.3b | 0.02683 | 1.57 |
| TGM3 | NM_003245.2 | 20p13d | 0.00651 | 1.56 |
| LILRA5 | NM_021250.2 | 19q13.42a | 0 | 1.55 |
| S100P | NM_005980.2 | 4p16.1f | 0.02683 | 1.55 |
| HIST2H2AA4 | NM_001040874.1 | 1q21.2a | 0.03779 | 1.52 |
| NCF4 | NM_000631.3 | 22q12.3d | 0.00183 | 1.51 |
| SIPA1L2 | NM_020808.3 | 1q42.2b | 0.01155 | 1.51 |
| SLC11A1 | NM_000578.3 | 2q35e | 0.01528 | 1.49 |
| SORL1 | NM_003105.3 | 11q24.1a | 0.00314 | 1.47 |
| FCGR2A | NM_021642.2 | 1q23.3a | 0.01332 | 1.47 |
| HSPA6 | NM_002155.3 | 1q23.3a | 0.02648 | 1.47 |
| ALOX5AP | NM_001629.2 | 13q12.3c | 0 | 1.45 |
| TREM1 | NM_018643.2 | 6p21.1g | 0 | 1.45 |
| CDA | NM_001785.2 | 1p36.12b | 0.00753 | 1.42 |
| FOLR3 | NM_000804.2 | 11q13.4a | 0.03086 | 1.42 |
| MSRB2 | NM_012228.2 | 10p12.2a | 0 | 1.40 |
| STX11 | NM_003764.2 | 6q24.2b | 0 | 1.40 |
| ORM1 | NM_000607.1 | 9q32d | 0.01332 | 1.39 |
| MBP | NM_001025101.1 | 18q23b | 0.04787 | 1.39 |
| ARG1 | NM_000045.2 | 6q23.2a | 0.04127 | 1.39 |
| LOC730278 | XM_001126471.1 |  | 0.01332 | 1.39 |
| MEGF9 | NM_001080497.1 | 9q33.2a | 0.01155 | 1.39 |
| C20ORF3 | NM_020531.2 | 20p11.21a | 0.02683 | 1.38 |
| HIST1H2BD | NM_138720.1 | 6p22.1d | 0.02013 | 1.38 |
| NFE2 | NM_006163.1 | 12q13.13f | 0.01155 | 1.38 |
| C19ORF59 | NM_174918.2 | 19p13.2e | 0.0256 | 1.38 |
| REPS2 | NM_004726.2 | Xp22.2 | 0.03106 | 1.37 |
| UBB | NM_018955.2 | 17p11.2i | 0.00948 | 1.37 |
| B4GALT5 | NM_004776.2 | 20q13.13d | 0.01332 | 1.36 |
| FRAT1 | NM_005479.2 | 10q24.1b | 0.03532 | 1.36 |
| FCGR3B | NM_000570.2 | 1q23.3b | 0.00753 | 1.35 |
| ATG2A | NM_015104.1 | 11q13.1b-q13.1c | 0 | 1.35 |
| ACSL1 | NM_001995.2 | 4q35.1e | 0.02013 | 1.35 |
| LOC347376 | XM_937928.1 | Xp11.22c | 0.03086 | 1.35 |
| S100A12 | NM_005621.1 | 1q21.3c | 0.02251 | 1.35 |
| CHST15 | NM_015892.2 | 10q26.13d | 0.03558 | 1.34 |
| SLC16A3 | NM_004207.2 | 17q25.3g | 0.00753 | 1.34 |
| TXN | NM_003329.2 | 9q31.3a | 0.00753 | 1.34 |
| FAM129A | NM_052966.2 | 1q25.3f | 0.024 | 1.34 |
| HSPA1A | NM_005345.4 | 6p21.33a | 0.0214 | 1.33 |
| LOC100133875 | XM_001720097.1 |  | 0.05023 | 1.33 |
| IGF2R | NM_000876.2 | 6q25.3f | 0.01332 | 1.33 |
| LOC730740 | XM_001128558.1 |  | 0 | 1.33 |
| HIST2H2BE | NM_003528.2 | 1q21.2a | 0.00753 | 1.33 |
| MTHFS | NM_006441.1 | 15q25.1b | 0 | 1.33 |
| LOC440093 | NM_001013699.1 | 12p11.21b | 0.01332 | 1.33 |
| HAL | NM_002108.2 | 12q23.1a | 0.02251 | 1.33 |
| NUP214 | NM_005085.2 | 9q34.13a | 0.01332 | 1.33 |
| BRI3 | NM_015379.3 | 7q21.3d | 0.03965 | 1.32 |
| SLC40A1 | NM_014585.4 | 2q32.2a | 0.02648 | 1.32 |
| MT2A | NM_005953.2 | 16q13b | 0 | 1.32 |
| C13ORF18 | NM_025113.1 | 13q14.12b | 0.00753 | 1.31 |
| IL10RB | NM_000628.3 | 21q22.11c | 0.03106 | 1.31 |
| SEC14L1 | NM_003003.2 | 17q25.2b | 0.04401 | 1.31 |
| FAR2 | NM_018099.3 | 12p11.22a | 0.01944 | 1.31 |
| S100A11 | NM_005620.1 | 1q21.3a | 0.01729 | 1.31 |
| P2RY13 | NM_023914.2 | 3q25.1c | 0.03779 | 1.31 |
| BRI3P1 | XR_015539.2 | 1p21.2a | 0.05023 | 1.31 |
| HSPA7 | NR_024151.1 |  | 0.05031 | 1.31 |
| BASP1 | NM_006317.3 | 5p15.1b | 0.00753 | 1.30 |
| DHRS9 | NM_005771.3 | 2q31.1a | 0.05023 | 1.30 |
| FTHL8 | NR_002203.1 | Xq28a | 0.01944 | 1.30 |
| RTN3 | NM_006054.2 | 11q13.1a | 0.01155 | 1.30 |
| AQP9 | NM_020980.2 | 15q22.1a | 0.02771 | 1.30 |
| ITPK1 | NM_014216.3 | 14q32.12b | 0.02865 | 1.30 |
| SERPINA1 | NM_001002235.1 | 14q32.13a | 0.02013 | 1.30 |
| ANXA3 | NM_005139.2 | 4q21.21a | 0.01729 | 1.29 |
| FRAT2 | NM_012083.2 | 10q24.1b | 0.03004 | 1.29 |
| SEPX1 | NM_016332.2 | 16p13.3e | 0.0214 | 1.29 |
| RNASE6 | NM_005615.4 | 14q11.2b | 0 | 1.29 |
| SIRPA | NM_080792.2 | 20p13d | 0.02648 | 1.29 |
| LST1 | NM_205840.1 | 6p21.33a | 0.01332 | 1.29 |
| LOC440926 | NR_002315.1 | 2q31.1g | 0.0256 | 1.29 |
| XPO6 | NM_015171.2 | 16p11.2e | 0.05044 | 1.29 |
| C7ORF59 | NM_001008395.2 | 7q22.1c | 0.00753 | 1.29 |
| FCAR | NM_133280.1 | 19q13.42b | 0.05008 | 1.28 |
| LOC392437 | XR_017149.2 | Xp21.2a | 0.03179 | 1.28 |
| TNFSF13B | NM_006573.3 | 13q33.3b | 0.02771 | 1.28 |
| C20ORF24 | NM_018840.2 | 20q11.23a | 0.04458 | 1.28 |
| IL18R1 | NM_003855.2 | 2q12.1a | 0.01332 | 1.28 |
| CAMP | NM_004345.3 | 3p21.31f | 0.02013 | 1.28 |
| RAB11FIP1 | NM_025151.3 | 8p12a | 0.04364 | 1.28 |
| GCA | NM_012198.2 | 2q24.2d | 0.01528 | 1.28 |
| LOC100130886 | XM_001714477.1 |  | 0.05023 | 1.28 |
| LOC100129905 | XM_001726007.1 | 11q23.3b | 0.00753 | 1.28 |
| ADM | NM_001124.1 | 11p15.4a | 0.05031 | 1.28 |
| HS.131087 | BX111162 |  | 0.04127 | 1.27 |
| ZNF738 | XR_040185.1 | 19p12d | 0.00753 | 1.27 |
| H3F3A | NM_002107.3 | 1q42.12c | 0 | 1.27 |
| SERPINB1 | NM_030666.2 | 6p25.2b | 0.04787 | 1.27 |
