# Supplementary material for: Dietary Methanol Regulates Human Gene Activity
Source: PLoS One. 2014 Jul 17;9(7):e102837. doi: 10.1371/journal.pone.0102837 (PMC4102594; doi:10.1371/journal.pone.0102837)
Supplement: Table S2 — The list of human down-regulated genes of volunteers after pectin intake. (DOC) [file pone.0102837.s012.doc]

| TargetID | Accession | Cytoband | q-value | Fold Change |
| --- | --- | --- | --- | --- |
| PRAGMIN | NM_001080826.1 | 8p23.1e | 0.01367 | -1.220 |
| CD36 | NM_000072.2 | 7q21.11c | 0.02719 | -1.270 |
| CCR7 | NM_001838.2 | 17q21.2a | 0 | -1.273 |
| C4ORF18 | NM_016613.4 | 4q32.1d | 0.01343 | -1.300 |
| MYC | NM_002467.3 | 8q24.21b | 0.01942 | -1.300 |
| LEF1 | NM_016269.2 | 4q25b | 0 | -1.301 |
| SESN1 | NM_014454.1 | 6q21f | 0 | -1.302 |
| PIK3IP1 | NM_052880.3 | 22q12.2c | 0 | -1.308 |
| CLC | NM_001828.4 | 19q13.2b | 0.0237 | -1.330 |
| KLF9 | NM_001206.2 | 9q21.11c | 0 | -1.342 |
| CD69 | NM_001781.1 | 12p13.31a | 0.00213 | -1.370 |
| HBA1 | NM_000558.3 | 16p13.3f | 0.01676 | -1.39 |
| IL7R | XM_937367.1 | 5p13.2c | 0.0041 | -1.41 |
| PRSS33 | NM_152891.2 | 16p13.3d | 0.0363 | -1.429 |
| HBA2 | NM_000517.3 | 16p13.3f | 0.00606 | -1.45 |
| HBB | NM_000518.4 | 11p15.4c | 0.01216 | -1.52 |
| DDIT4 | NM_019058.2 | 10q22.1f | 0.00438 | -1.54 |
