# Supplementary material for: Dietary Methanol Regulates Human Gene Activity
Source: PLoS One. 2014 Jul 17;9(7):e102837. doi: 10.1371/journal.pone.0102837 (PMC4102594; doi:10.1371/journal.pone.0102837)
Supplement: Table S3 — The list of down-regulated genes in intersection of the Venn diagram circles presented in Figure 5B . (DOC) [file pone.0102837.s013.doc]

| TargetID | ACCESSION | Fold Change | q-val[i] |
| --- | --- | --- | --- |
| ZFP330 | NM_145600.1 | -4.538 | 0 |
| HBA-A1 | NM_008218.2 | -3.409 | 0 |
| SERPINA3N | NM_009252.2 | -3.315 | 0 |
| HBB-B2 | NM_016956.2 | -2.444 | 0 |
| PRDX2 | NM_011563.2 | -2.417 | 0 |
| NRSN2 | NM_001009948.1 | -1.925 | 0 |
| PRDX2 | NM_011563.2 | -1.863 | 0 |
| SCOC | NM_001039137.2 | -1.807 | 0 |
| TMEM68 | NM_028097.3 | -1.750 | 0 |
| CLIP4 | NM_030179.2 | -1.737 | 0 |
| ATG3 | NM_026402.3 | -1.671 | 0 |
| MID1 | NM_183151.1 | -1.644 | 0 |
| BC056474 | NM_001001493.2 | -1.496 | 0 |
| NUDT19 | NM_033080.2 | -1.469 | 0 |
| HBB-B1 | NM_008220.3 | -1.452 | 0 |
| XLR4A | NM_001081642.1 | -1.427 | 0 |
| GNB1 | NM_008142.3 | -1.416 | 0.04 |
| EXOC7 | NM_016857.1 | -1.360 | 0.02564 |
| 8430408G22RIK | NM_145980.1 | -1.339 | 0 |
| D14ERTD449E | NM_025311.1 | -1.328 | 0 |
| 9030607L17RIK | NM_027829.3 | -1.327 | 0.02564 |
| P4HA1 | NM_011030.1 | -1.326 | 0.04 |
| H2-K1 | NM_001001892.1 | -1.310 | 0.04 |
| GUCY1A3 | NM_021896.4 | -1.309 | 0.02564 |
