# Supplementary material for: Dietary Methanol Regulates Human Gene Activity
Source: PLoS One. 2014 Jul 17;9(7):e102837. doi: 10.1371/journal.pone.0102837 (PMC4102594; doi:10.1371/journal.pone.0102837)
Supplement: Table S4 — The list of up-regulated genes in intersection of the Venn diagram circles presented in Figure 5B . (DOC) [file pone.0102837.s014.doc]

| TargetID | ACCESSION | Fold Change | q-val[i] |
| --- | --- | --- | --- |
| NUDT11 | NM_021431.2 | 6.188 | 0 |
| SERPINI1 | NM_009250.1 | 4.120 | 0 |
| TESK1 | NM_011571.2 | 3.563 | 0 |
| SLC25A18 | NM_001081048.1 | 3.199 | 0 |
| CPLX2 | NM_009946.2 | 2.646 | 0 |
| LRRC57 | NM_025657.2 | 2.567 | 0 |
| SLC35E3 | NM_029875.2 | 2.085 | 0 |
| CD6 | NM_009852.3 | 2.001 | 0 |
| PYGB | NM_153781.1 | 1.899 | 0 |
| SMYD3 | NM_027188.3 | 1.741 | 0 |
| HSPB6 | NM_001012401.1 | 1.624 | 0.04167 |
| B3GALNT1 | NM_020026.2 | 1.618 | 0 |
| MYL1 | NM_021285.1 | 1.606 | 0.04167 |
| CDKAL1 | NM_144536.1 | 1.591 | 0 |
| ABHD1 | NR_003522.1 | 1.579 | 0.02439 |
| MFSD2 | NM_029662.1 | 1.369 | 0 |
| ABHD1 | NM_021304.2 | 1.354 | 0 |
| SLPI | NM_011414.2 | 1.298 | 0.02439 |
