# Supplementary material for: Dietary Methanol Regulates Human Gene Activity
Source: PLoS One. 2014 Jul 17;9(7):e102837. doi: 10.1371/journal.pone.0102837 (PMC4102594; doi:10.1371/journal.pone.0102837)
Supplement: Table S5 — Oligonucleotides used for qPCR. (DOC) [file pone.0102837.s015.doc]

| **Gene** | **Forward primer** | **Reverse primer** | **PCR product length, bp** | **Ta, ˚C** |
| --- | --- | --- | --- | --- |
| h_Alas2 | AAAGGCTGGAGGAGATTC | CTTAGGCTGACTGAGACC | 171 | 54.8 |
| h_DDIT4 | GAGGTCGTGGAGGTGGTTTG | ACATTCATGCGTCTGTAAGATAGC | 139 | 53.4 |
| h_EPB42 | GAGCAGGAGAAGGCAGTG | GCGGTGAGTCTAAGGAAGG | 194 | 56.3 |
| h_GAPDH | TCTGGTAAAGTGGATATTGTTG | CCTGGAAGATGGTGATGG | 187 | 54 |
| h_GYPA | TACGCACAAACGGGACAC | CCAATAACACCAGCCATCACC | 177 | 53.3 |
| h_HBA1 | CTGCCGACAAGACCAACG | GGGAAGGACAGGAACATC | 100 | 56.4 |
| h_HBB | AGCAACCTCAAACAGACAC | CTCACCACCAACTTCATCC | 101 | 53.9 |
| h_MME | TCGTTGACTGGTGGACTC | GATAGGCTCTGTATGCTTGAC | 180 | 53.3 |
| h_RPL32 | CATCTCCTTCTCGGCATCA | AACCCTGTTGTCAATGCCTC | 152 | 56 |
| h_Snx27 | ACGGCATTCTCTGTGACTTC | AACCATATTCCTACTACTCCTTCC | 163 | 56 |
| h_SORL | GACTTCCGACTCACAATC | TTCACATCCTCAGACACC | 173 | 54.6 |
| h_SPTA1 | CAGTGATGAAATAGAGAATGC | AAGTAGGAATTGGTGAAGC | 192 | 53.2 |
| m_Alas2 | CCAAGGCATTCGCAACAG | GAAGGTCAGGGCTCCATAC | 199 | 55.2 |
| m_EPB4 | AACTCCCAAACAACCCTCAAC | ATACCAACCAACCAACCAACC | 164 | 52.8 |
| m_GYPA | GTCTTCCCGTGTTCTTCC | GGCTGTGAGTGTCCTTAC | 117 | 52.5 |
| m_HBA-A1 | GGGGAAGACAAAAGCAACATC | ACATCAAAGTGAGGGAAGTAGG | 134 | 55 |
| m_HBB | CGATGAAGTTGGTGGTGAG | ATGATAGCAGAGGCAGAGG | 105 | 54.2 |
| m_RPL32 | GGCACCAGTCAGACCGATATG | CCTTCTCCGCACCCTGTTG | 80 | 56 |
| m_SPTA1 | ACAAGGAGTCAGAGAATATCAAG | ACATAGTCGTAGCCAGCAG | 196 | 54.6 |
